# Experimental factors that impact Ca_V_1.2 channel pharmacology—Effects of recording temperature, charge carrier, and quantification of drug effects on the step and ramp currents elicited by the “step-step-ramp” voltage protocol

**DOI:** 10.1371/journal.pone.0276995

**Published:** 2022-11-23

**Authors:** Ming Ren, Aaron L. Randolph, Claudia Alvarez-Baron, Donglin Guo, Phu N. Tran, Nicolas Thiebaud, Jiansong Sheng, Jun Zhao, Wendy W. Wu

**Affiliations:** 1 Division of Applied Regulatory Science, Office of Clinical Pharmacology, Center for Drug Evaluation and Research, US Food and Drug Administration, Silver Spring, Maryland, United States of America; 2 Nanion Technologies Inc, Livingston, New Jersey, United States of America; 3 Division of Cardiology and Nephrology, Office of Cardiology, Hematology, Endocrinology and Nephrology, Office of New Drugs, Center for Drug Evaluation and Research, US Food and Drug Administration, Silver Spring, Maryland, United States of America; 4 Division of Immunology and Hematology Devices, Center for Devices and Radiological Health, US Food and Drug Administration. Silver Spring, Maryland, United States of America; 5 Vertex Pharmaceuticals (Europe) Ltd, Abingdon, Oxfordshire, United Kingdom; 6 CiPA Lab, Gaithersburg, Maryland, United States of America; The Open University, UNITED KINGDOM

## Abstract

**Background and purpose:**

Ca_V_1.2 channels contribute to action potential upstroke in pacemaker cells, plateau potential in working myocytes, and initiate excitation-contraction coupling. Understanding drug action on Ca_V_1.2 channels may inform potential impact on cardiac function. However, literature shows large degrees of variability between Ca_V_1.2 pharmacology generated by different laboratories, casting doubt regarding the utility of these data to predict or interpret clinical outcomes. This study examined experimental factors that may impact Ca_V_1.2 pharmacology.

**Experimental approach:**

Whole cell recordings were made on Ca_V_1.2 overexpression cells. Current was evoked using a “step-step-ramp” waveform that elicited a step and a ramp current. Experimental factors examined were: 1) near physiological vs. room temperature for recording, 2) drug inhibition of the step vs. the ramp current, and 3) Ca^2+^ vs. Ba^2+^ as the charge carrier. Eight drugs were studied.

**Key results:**

Ca_V_1.2 current exhibited prominent rundown, exquisite temperature sensitivity, and required a high degree of series resistance compensation to optimize voltage control. Temperature-dependent effects were examined for verapamil and methadone. Verapamil’s block potency shifted by up to 4X between room to near physiological temperature. Methadone exhibited facilitatory and inhibitory effects at near physiological temperature, and only inhibitory effect at room temperature. Most drugs inhibited the ramp current more potently than the step current—a preference enhanced when Ba^2+^ was the charge carrier. The slopes of the concentration-inhibition relationships for many drugs were shallow, temperature-dependent, and differed between the step and the ramp current.

**Conclusions and implications:**

All experimental factors examined affected Ca_V_1.2 pharmacology. In addition, whole cell Ca_V_1.2 current characteristics—rundown, temperature sensitivity, and impact of series resistance—are also factors that can impact pharmacology. Drug effects on Ca_V_1.2 channels appear more complex than simple pore block mechanism. Normalizing laboratory-specific approaches is key to improve inter-laboratory data reproducibility. Releasing original electrophysiology records is essential to promote transparency and enable the independent evaluation of data quality.

## Introduction

Ca_V_1.2 channels in the heart mediate L-type Ca^2+^ current that contributes to Ca^2+^-dependent action potentials (APs) in the pacemaker cells of the sinoatrial and atrioventricular nodes, the plateau phase of the AP in the working myocytes, and triggers cardiac excitation-contraction coupling [1]. Drugs that reduce Ca_V_1.2 channel activity slow heart rate, shorten AP duration in atrial and ventricular myocytes, and decrease contractile force. Ca_V_1.2 channel agonists have not been used in humans. Nonetheless, studies have shown that pharmacologically enhancing L-type Ca^2+^ current can produce delayed repolarization and ventricular arrhythmias [2, 3]. Therefore, understanding drug effects on Ca_V_1.2 channels may provide insights regarding a drug’s impact on cardiac function. Indeed, a survey of nonclinical safety assessment of proarrhythmia risk used by the pharmaceutical industry found that during early drug discovery, patch clamp characterization of a drug candidate’s effect on Ca_V_1.2 channels is routinely performed, with frequency second only to the hERG assay [4].

Despite of the potential of Ca_V_1.2 data to inform drug effect in the clinical setting, leveraging *in vitro* patch clamp results has been challenging for drug regulators. Because of the high inter-laboratory data variability reported in the literature, a major concern is that conclusions based on these data are laboratory-dependent. Inter-laboratory data variability is often ascribed to the use of different voltage protocols and stimulation frequencies. However, results from two recent publications suggest that additional factors may be involved [5, 6]. In these studies, Ca_V_1.2 current was recorded using overexpression cell lines, and evoked from the same holding potential, at the same frequency (0.1 Hz), using either a ventricular AP waveform [5] or a ventricular AP-like “step-step-ramp” waveform [6]. Nonetheless, surprisingly different inhibitory potencies were obtained for the same drugs, with difference up to 1743X reported (Table 1). These results underscore the need to understand the conduct and design of Ca_V_1.2 experiments—how each experimental factor may translate into differences in drug effect, especially if these *in vitro* results are to be used in decision-making regarding risk prediction or mechanistic interpretation of clinical outcomes.

**Table 1:** IC_50_ differences for Ca_V_1.2 channel block reported by Crumb et al. 2016 [5] and Li et al. 2018 [6].

	Crumb et al. 2016 [5]		Li et al. 2018 [6]		Ratio (max-to-min)	Log P
	IC_50_ (μM)	n_H_	IC_50_ (μM)	n_H_		
**Bepridil**	2.8	0.6	638	4.6	228	5.49
**Chlorpromazine**	8.2	0.8	6.35	2	1.3	4.54
**Diltiazem**	0.1	0.7	31.6	1.2	316	2.73
**Ondansetron**	22.6	0.8	9310	0.2	412	2.35
**Terfenadine**	0.7	0.7	1220	5.2	1743	6.48
**Verapamil**	0.2	1.1	11.2	0.8	56	5.04

Comparison of the abovementioned publications revealed several differences in the Ca_V_1.2 experimental conduct and design. One study used a manual patch clamp system, recorded cells at near physiological temperature (PT) using Ba^2+^ as the charge carrier, and quantified drug effect on the inward current associated with the repolarizing phase of the AP [5]. The other used an automated patch clamp system, recorded cells at ambient temperature using Ca^2+^ as the charge carrier, and quantified drug effect on the inward current triggered by the initial voltage step [6]. Recording temperature [7, 8] and charge carrier [9–12] are known to affect block potencies for some drugs on Ca_V_1.2 channels in overexpression cells and L-type channels in native myocytes. In addition, measuring drug effects on Ca_V_1.2 current evoked at different time points following the initial depolarization and associated with different membrane voltages (i.e., current resulting from different channel state, due to activation [6] or reactivation following recovery from inactivation [5]), as was done in these two studies is also a likely source of data variability, given that many drugs are known to block Ca_V_1.2 and L-type Ca^2+^ channels in a state-dependent manner [13–17].

Using manual whole cell patch clamp method to record cells stably expressing hCa_V_1.2α, β_2_, and α_2_δ_1_ subunits, the present study was conducted to examine Ca_V_1.2 current characteristics and the impact of the recording temperature, charge carrier, and current region where drug effects were quantified on pharmacology. Results show that Ca_V_1.2 current exhibits prominent rundown following whole cell formation, was exquisitely temperature sensitive, and required a high degree of series resistance compensation to optimize voltage control. These characteristics meant that drug-independent changes in the current amplitude can be anticipated during long lasting pharmacology experiments, and laboratory-specific practices to deal with these changes can be sources of data variability. In addition, all experimental factors examined affected drug potency estimations, with drug-specific magnitude and direction of change. For Ca_V_1.2 data intended to support risk prediction or clinical interpretation, normalizing laboratory-specific practices is essential to promote data reproducibility across laboratories—a pivotal step toward engendering confidence amongst regulators for applying these *in vitro* data in the decision-making process. To support data transparency, the original electrophysiology records, detailed cell culture procedure, and supplemental materials for the present study are available for download at: https://osf.io/g3msb/.

## Methods

### Cells

CHO cells stably transfected with hCa_v_1.2α, β_2_, and α_2_δ_1_ subunits (Charles River Laboratory; CT3004) were cultured at 5% CO_2_ and 37°C, following passage in Ham’s F12 media with L-glutamine nutrient mixture (Gibco #11765054) supplemented with 10% tetracycline-screened fetal bovine serum (FBS) (Cytiva Hyclone SH30071.03T) and the following cell selection reagents: Blasticidin (0.01 mg/mL; Gibco #A1113903), Geneticin (G418; 0.25 mg/mL; Sigma G8168), Hygromycin (0.25 mg/mL; Sigma H0654), and Zeocin (0.40 mg/mL; Invitrogen #46–0509). Cells were seeded at low density and kept in culture for 4–7 days before seeding on glass coverslips for electrophysiology use. By the time cells were detached for seeding, they were fully confluent. Twenty-four to 48 hours prior to recording, cultures were washed with DPBS without Ca^2+^ or Mg^2+^ (Gibco #14190144), and then detached by applying Accutase (Sigma A6964) for 2 minutes. Cell suspensions of 30,000–40,000 cells/mL were added to 35 mm petri dishes containing 12 mm glass coverslips, in Ham’s F12 media containing only 10% FBS. Cells were kept at 5% CO_2_ and 37°C until recording. For this cell line, the expressions of β_2_ and α_2_δ_1_ subunits were constitutive, while that of the pore-forming α subunit required tetracycline induction. Three protocols were used for tetracycline induction to accommodate staff schedule. For the first protocol, cells were seeded late in the afternoon the day prior to recording. On the next day, 16–20 hours after seeding, 2.5 μg/mL tetracycline (Sigma T7660) was added to the petri dishes for 4 hours prior to recording. For the second protocol, cells seeded the day before recording were allowed to attach to glass coverslips for ~6 hours, and 0.5 μg/mL tetracycline was added for overnight induction (typically 16–20 hours prior to recording on the following day). For the third protocol, cells were seeded 4 days prior to recording. The day before recordings, cells were fully detached and seeded in media containing 1 μg/mL tetracycline. On the day of the recording, cells were detached again and seeded on glass coverslips. Regarding the first two protocols, after induction of the α subunit cells adopted a very flat morphology, rendering patching and maintaining long lasting recordings challenging. Cells generated using the third protocol were easier to patch due to the more rounded morphology. The use of different cell culture procedures did not impact pharmacology in this study. The amplitude of Ca_V_1.2 current was dependent on both the amount of tetracycline used and the duration of induction.

### Electrophysiology

Voltage clamp recordings were made with Multiclamp 700B amplifier (Molecular Devices, CA) and digitized using a Digidata 1550B (Molecular Devices, CA) interface and the pClamp 10 software (Molecular Devices, CA). Glass coverslips with cells were placed in a recording chamber mounted on an inverted (Zeiss Axiovert 135TV or A1) or an upright microscope (Zeiss AxioExaminer D1), and the recording chamber was continuously perfused using a gravity-fed perfusion system, with an external solution flowing at a rate of 1.5–3 mL/min. Temperature of the recording solution was elevated using a dual channel temperature controller. Two controller models were used: 1) TC2BIP from Cell MicroControls, which elevated the solution temperature with an inline solution heater, and maintained the bath temperature by heating the ITO-coated glass coverslip which formed the bottom of the recording chamber; and 2) TC-344C from Warner Instruments, which elevated the solution temperature with an inline solution heater, and maintained the bath temperature by heating up the anodized aluminum platform (PH-1) that supported the edge of the plastic recording chamber. Two differences were noted regarding these two controller models: 1) at near 37°C, ~2°C difference between the inflow and center and the recording chamber was observed for TC-344C; and 2) TC2BIP provided more stable temperature control near 37ºC than TC-344C, even when the flow rate changed. Bath temperature in the recording chamber was recorded using a thermistor placed in the bath throughout the experiment. Whole-cell current was recorded at near physiological temperature (PT; 37 ± 2°C) or room temperature (RT; 23 ± 1°C). While most of the recordings in this study aimed at 37°C, the term “near PT” was used to acknowledge the few degrees of temperature fluctuations that occurred during the experiments.

The internal solution contained (in mM): 120 aspartic acid, 120 CsOH, 10 CsCl, 10 EGTA, 5 MgATP, 0.4 TrisGTP, 10 HEPES; pH adjusted to 7.4 with 5M CsOH; ~290 mOsM. When Ca^2+^ was used as the charge carrier, the external solution contained (in mM): 137 NaCl, 4 KCl, 1.8 CaCl_2_, 1 MgCl_2_, 10 HEPES, 10 glucose; pH adjusted to 7.4 with 5M NaOH; ~290 mOsM. When Ba^2+^ was used as the charge carrier, the external solution contained (in mM): 137 NaCl, 4 KCl, 4 BaCl_2_, 1 MgCl_2_, 10 HEPES, 10 glucose; pH adjusted to 7.4 with 5M NaOH; ~290 mOsM. Recording electrodes were made by pulling borosilicate glass pipettes (BF150-86-10; Sutter Instrument, CA) with a P97 micropipette puller (Sutter Instruments, CA), and had tip resistances in the range of 1.5–2.5 MΩ when filled with the internal solution. The voltage command values were corrected for the 17 mV liquid junction potential (LJP) that resulted from using the above internal solution and Ca^2+^-containing external solution at 37°C, estimated using the PClamp 10 software. Given that voltage sensed by the membrane equals to voltage at the pipette minus the LJP (or V_m_ = V_pipette_ − V_LJP_), to hold the cell at -80 mV, the input voltage was set at -63 mV. The 17 mV LJP correction was also applied to RT recordings using Ca^2+^ as the charge carrier (LJP at 23°C was estimated to be 16 mV), and to near PT recordings using Ba^2+^ as the charge carrier (LJP at 37°C was estimated to be 17 mV). The voltage waveform used was as follows: from a holding potential of -80 mV, the cell was hyperpolarized to -90 mV for 100 ms, repolarized to -80 mV for 100 ms, depolarized to 0 mV for 40 ms, further depolarized to +30 mV for 200 ms, and finally ramped down to -80 mV in 100 ms (-1.1 V/s). This waveform, modified from that used by Li et al., 2018 [6], was chosen as it evoked an inward current peak at the 0 mV step (where Li et al., 2018 characterized drug effects at) and another one at the repolarizing ramp that reflects the channel state of what Crumb et al., 2016 studied [5]. The use of this voltage protocol therefore permitted a direct comparison of drug effects at the step and the ramp current that were separated in time and associated with different membrane voltages/channel states. The voltage waveform was delivered at 5 s intervals or 0.2 Hz. Signals were filtered at 3 or 10 kHz and sampled at 10 kHz. Whole cell capacitance was neutralized. Series resistance (R_s_) was measured approximately 2 minutes following whole cell formation, after signs of membrane resealing were no longer evident, using the membrane test function of the pClamp 10 software. R_s_ was electronically compensated at 80%. The MultiClamp 700B R_s_ compensation bandwidth control replaces the “lag” control on earlier Axon amplifier series: Bandwidth = 1 / (2 * π * Lag). This study used the default R_s_ correction bandwidth of 1.02 kHz, which is equivalent to a lag value of 156 μs. For the pharmacology dataset in this manuscript, R_s_ was 4.5 ± 0.1 MΩ (± SEM; n = 295; RT and near PT data combined); whole cell capacitance was 35.2 ± 0.9 pF (n = 294; value from one cell was not captured).

Ca_V_1.2 current showed pronounced rundown in whole cell configuration (see “Results and discussion”). To study drug effects, after Ca_V_1.2 current reached a quasi-steady state level in the control solution, drug solution was perfused as the recording continued. Depending on the cell quality and stability achieved in the control solution, 1 to 2 drug concentrations were tested per cell. Verapamil at 100 μM was used as a full blocker and was applied at the end of the recordings whenever possible.

### Drugs

Naloxone hydrochloride (0599), tolterodine L-tartrate (3761), and diltiazem hydrochloride (0685) were purchased from Tocris Bioscience. Buprenorphine hydrochloride (B9275, USDEA C-III), (±)-methadone hydrochloride (M0267, USDEA C-II), naltrexone hydrochloride (N3136), (±)-verapamil hydrochloride (V4629), and DMSO (D8418) were purchased from Sigma-Aldrich. Norbuprenorphine hydrochloride (USDEA C-II) was purchased from Noramco. Stock solutions of naloxone, naltrexone, and verapamil were dissolved in milliQ water. Stock solutions of methadone, buprenorphine, norbuprenorphine, diltiazem, and tolterodine were dissolved in DMSO. When DMSO was used as a solvent, the % of DMSO exposed to cells was ≤0.3%. Aliquoted stock solutions were stored at -20°C until the day of experiments and were diluted to specific test concentrations in the external solution.

### Data analysis and reporting

Data analysis and curve fitting were done in Clampfit 10.6 (Molecular Devices, CA) and Igor Pro 8.0 (WaveMetrics). Two offline methods were used to isolate Ca_V_1.2 current from the total inward current. The first method was the passive current (I_passive_) subtraction. Ohm’s law was used to calculate the resting input resistance (R_input_) for each current trace:

$${\text{R}}_{\text{input}}=\left({\text{V}}_{-90\;\text{mV}}-{\text{V}}_{-80\;\text{mV}}\right)/\left({\text{I}}_{-90\;\text{mV}}-{\text{I}}_{-80\;\text{mV}}\right)$$

Here I_-80 mV_ refers to the current measured at the holding potential of -80 mV (V_-80 mV_), and I_-90 mV_ the current measured during hyperpolarizing step to -90 mV (V_-90 mV_). I_passive_ was defined to exhibit linear current-voltage (I-V) relationship. Therefore, assuming that R_input_ was constant across all voltages, I_passive_ was calculated using the following equation:

$${\text{I}}_{\text{passive}}={\text{I}}_{-80\;\text{mV}}+\left(\text{V}-{\text{V}}_{-80\;\text{mV}}\right)/{\text{R}}_{\text{input}}$$

Here V refers to any voltage within the “step-step-ramp” protocol. Using the custom macros written for Igor Pro, I_passive_ was calculated for each current trace and then subtracted from that trace to yield Ca_V_1.2 current.

The second current subtraction method was verapamil subtraction. At positive membrane potentials, a population of cells exhibited a non-linear outward current that was not removed by I_passive_ subtraction. This outward current was most notable at the +30 mV step and the adjoining repolarizing ramp section. For these cells, the residual current trace in the presence of 100 μM verapamil was subtracted from all current traces to isolate Ca_V_1.2 current. Verapamil subtraction was performed by averaging several traces recorded in verapamil that exhibited full inhibition of the ramp current, and then subtracting this averaged trace from all recorded current traces. For the methadone experiments, some cells did not receive 100 μM verapamil. Nonetheless, complete elimination of the ramp current was achieved by 100 and 300 μM methadone. In these cases, several traces following full ramp current inhibition by methadone were averaged and then subtracted from all recorded traces to isolate Ca_V_1.2 current. S1 Fig illustrates these two subtraction methods and the phenotype of cells to which each was applied. Of note, even when the ramp current was completely eliminated by verapamil or methadone, a sizable portion of the 0 mV step current remained (see “Results and discussions”). Therefore, the step current was always quantified from I_passive_-subtracted traces. The ramp current was quantified using I_passive_-subtracted traces when there was no overt outward current at the positive membrane potentials or using verapamil-subtracted (or methadone-subtracted) traces when there was overt outward current, and the recording showed no time-dependent changes in the passive membrane properties, inferred from stability of R_input_ and I_-80 mV_.

From the I_passive_- or verapamil-subtracted current traces, the step and the ramp current were measured as the most negative inward current at the 0 mV step and the entire repolarizing ramp, respectively. Fractional inhibition by the tested drug for each cell was calculated with the following equation:

$$\text{Fractional}\;\text{inhibition}=1-\left({\text{I}}_{\text{drug}}/{\text{I}}_{\text{control}}\right)$$

Here I_drug_ is the averaged current amplitude from the last 10 traces recorded in the drug concentration, and I_control_ is the averaged current amplitude from the last 10 traces recorded in the control solution. Fractional inhibition values for individual cells were plotted against concentrations tested to yield concentration-inhibition graphs, and individual data points were fit with the Hill equation to estimate drug potency:

$$\text{Fractional}\;\text{inhibition}=1/(1+{\left({\text{IC}}_{50}/\left[\text{drug}\right]\right)}^{\text{n}}{}^{{}_{\text{H}}})$$

Here IC_50_ is the concentration that inhibited 50% of the current, [drug] is the drug concentration, and n_H_ is the Hill coefficient. The upper and lower 95% confidence interval (CI) bands were also plotted in the concentration-inhibition graphs to demonstrate uncertainty of the fit parameters. Except for the IC_50_ and n_H_ values, all data are presented as mean ± SEM.

IC_50_s of buprenorphine, norbuprenorpine, methadone, naltrexone, and naloxone on the ramp current studied in external Ca^2+^ and at 37ºC were published previously [18]. The IC_50_ values there differed slightly from those presented in this manuscript because they were estimated by fitting the averaged fractional inhibition values (instead of individual cells’ values) at different concentrations.

A t-test with equal variance was performed to compare the extent of Ca^2+^ and Ba^2+^ current rundown, using Prism version 8.4 (GraphPad, CA).

## Results and discussion

### Rundown of Ca_V_1.2 current in external Ca^2+^ or Ba^2+^ at near PT

**Fig 1:**
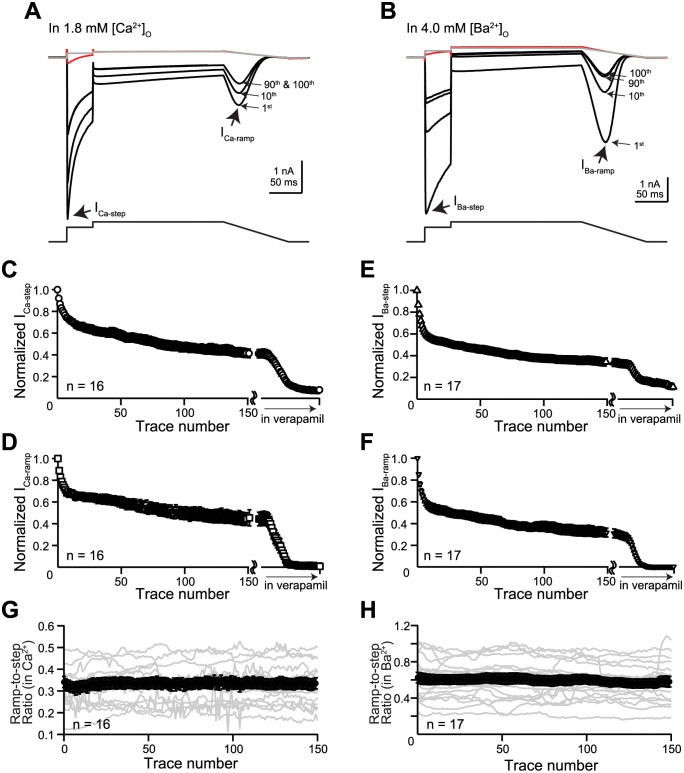
Rundown of Ca_V_1.2 channel activity at near PT. **A, B**. *Top*, representative current traces from 2 cells recorded in either external Ca^2+^ **(A)** or Ba^2+^ **(B)**. Cell ID for **(A)** was 19327000; for **(B)**, 19404005. Black traces reflect recordings obtained in the control solution, and the 1^st^, 10^th^, 90^th^, and 100^th^ traces recorded after ~2 minutes of whole cell dialysis are shown. Red traces reflect recordings obtained following application of 100 μM verapamil, after steady state inhibition was achieved. I_passive_ traces, shown in gray, were calculated using R_input_ derived from the verapamil traces. *Bottom*, the voltage protocol used. **C, D)** Summary time course plots of normalized I_Ca-step_ **(C)** and I_Ca-ramp_ **(D)** in the control solution and following verapamil application (n = 16). Data points are shown as mean ± sem. Verapamil application did not start at the same time for every cell. Therefore, the X-axes show a break after trace 150 to synchronize the data points obtained following verapamil application. **E, F)** Summary time course plots of normalized I_Ba-step_ **(E)** and I_Ba-ramp_ **(F)** in the control solution and following verapamil perfusion (n = 17). **G, H)** Ratio of I_Ca-ramp_-to-I_Ca-step_ **(G)** or I_Ba-ramp_-to-I_Ba-step_ **(H)** for traces acquired in the control solution. Data for individual cells are shown as light gray lines. Mean ± sem are shown as black symbols plus error bars.

Fig 1A and 1B show Ca_V_1.2 current recorded in external Ca^2+^ and Ba^2+^, respectively. The maximal peak current evoked by the 0 mV step is referred to as I_Ca-step_ or I_Ba-step_ depending on the charge carrier used; the maximal ramp current evoked by the repolarizing ramp, I_Ca-ramp_ or I_Ba-ramp_. Fig 1C–1F show the time course plots of normalized current amplitudes recorded in the control solution and following verapamil (100 μM) application. After whole cell formation, Ca_V_1.2 current showed prominent rundown regardless of which charge carrier was used, and in most cells eventually reached a quasi-steady state level. Current rundown was seen in every cell and was characterized by a fast phase followed by a slower phase. The normalized I_Ca-step_ at the 150^th^ trace was 0.42 ± 0.04 relative to the 1^st^ trace (Fig 1C); the normalized I_Ca-ramp_, 0.44 ± 0.06 (Fig 1D). The normalized I_Ba-step_ was 0.34 ± 0.04 (Fig 1E); the normalized I_Ba-ramp_, 0.32 ± 0.04 (Fig 1F). To assess whether the extent of rundown was different between the Ca^2+^ and the Ba^2+^ current, a basic statistical analysis of the differences in the amplitude of the 150^th^ trace relative to the first trace was performed. A t-test with equal variance revealed no significant difference between I_Ca-step_ and I_Ba-step_ and between I_Ca-ramp_ and I_Ba-ramp_ (p > 0.05). Fig 1G and 1H show the time course of the ratios of ramp-to-step current for traces acquired in the control solution. For Ca^2+^ current, the averaged ratio was 0.33 ± 0.03; for Ba^2+^ current, 0.60 ± 0.05. The larger ratio obtained in Ba^2+^ is consistent with the removal of Ca^2+^-dependent inactivation [19, 20]. These ratios remained constant throughout the control recording despite current rundown, suggesting that rundown reflects a progressive loss in the available channels. Rundown assessed using the present voltage protocol at 0.2 Hz was not attributed to intracellular Ca^2+^ accumulation, since it occurred to a similar degree in external Ba^2+^.

Application of verapamil nearly eliminated the ramp current (Fig 1D and 1F) but not the step current (Fig 1C and 1E). Relative to the 150^th^ trace recorded in the control solution, the residual I_Ca-step_ in verapamil was 20 ± 2%, while that of I_Ca-ramp_ was 4 ± 1%. Likewise, the residual I_Ba-step_ in verapamil was 32 ± 4%, while that of I_Ba-ramp_ was 2 ± 0%. Therefore, amplitude of the step current was always quantified using I_passive_-subtracted traces, not verapamil-subtracted traces (see “Methods”).

Rundown of Ca_V_1.2 current in whole cell configuration is a widely observed phenomenon. Since rundown leads to overestimation of fractional inhibition, laboratory-specific tolerance regarding the rate of rundown and practices to correct rundown can introduce variable degrees of imprecision into drug potency estimation. The diverse practices are exemplified by the two publications that motivated the present study. Crumb et al., 2016 did not correct for rundown, stated that cells with >20% rundown were discarded, but did not define how many current traces the 20% calculation was based [5]. Li et al., 2018 used perforated population recording that presumably reduced rundown, yet still corrected for rundown in the calculation of drug inhibition [6]. Even if the rates of Ca_V_1.2 current rundown were similar between the two publications, these different practices alone would lead to different drug potency estimations. Experience from this laboratory indicates that the rate of rundown, and whether current can reach a quasi-steady state in the control solution for pharmacology experiments are functions of the cell line used and cell culture conditions, and therefore unlikely to be the same across studies. Using the same Ca^2+^-external solution, internal solution, and voltage protocol, two additional Ca_V_1.2 cell lines that expressed the same channel subunits were tested, and both cell lines showed near complete loss of Ca_V_1.2 current at near PT with time, without reaching a quasi-steady state level (S2 Fig). Can Ca_V_1.2 current rundown under whole cell mode be prevented? Previous studies suggest that rundown of cardiac L-type Ca^2+^ channel activity reflects channel dephosphorylation. This conclusion is based on evidence from native myocytes that manipulating protein kinase A (PKA)-mediated phosphorylation [21], protein phosphatase activity [21], and increasing the intracellular level of ATP or cyclic AMP—molecules that enhance PKA-mediated phosphorylation [22] all led to expected changes in Ca^2+^ channel activity. In the present study, inclusion of 5 mM MgATP and 0.4 mM TrisGTP in the internal solution did not prevent current rundown in the three cell lines tested. Therefore, rundown in whole cell configuration could not be prevented by simply supplying ATP.

### Temperature sensitivity of Ca_V_1.2 current

**Fig 2:**
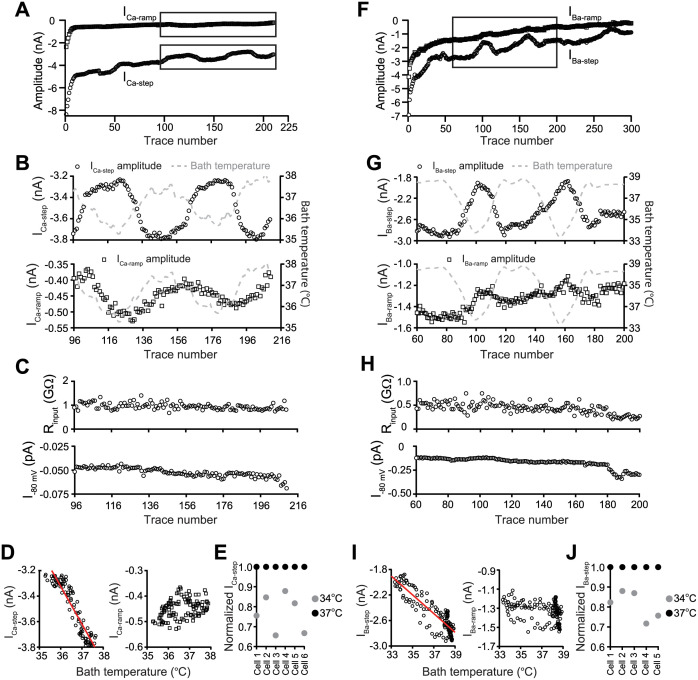
Effects of recording temperature on Ca_V_1.2 channel activity. Cell ID for **(A)** through **(D)** was 19318007. Recordings were obtained in external Ca^2+^. **A)** Time course plots of I_Ca-step_ (open circle) and I_Ca-ramp_ (open square) for the entire experiment. The boxed regions were expanded and shown in (**B**). **B)** *Top*, time course plots of I_Ca-step_ from traces 96 to 216 and bath temperature (gray dotted line). Current amplitudes were corrected for rundown. This could be done since rundown correction was performed on the traces used for the fit. To estimate rundown, data points in **(B)** were fit with a linear function to yield a slope of 0.00495 nA/trace. This amount of current loss was then added back to the original I_Ca-step_ amplitudes. *Bottom*, time course plots of I_Ca-ramp_ and bath temperature. Current amplitudes were also corrected for run rundown (slope = 0.00146 nA/trace). **C)** R_input_ (*top*) and I_-80 mV_ (*bottom*). **D)** *Left*, rundown-corrected I_Ca-step_ vs. bath temperature. These data points were fit with a linear function, yielding a slope of -0.295 nA/°C and a Y-intercept of 7.299 nA (r = -0.94). *Right*, rundown-corrected I_Ca-ramp_ vs. bath temperature (r = 0.38). **E)** Calculated and normalized I_Ca-step_ at 37°C (black circle) and 34°C (gray circle). Cell ID for **(F)** to **(J)** was 07_07_0008. Recordings were obtained in external Ba^2+^. **F)** Time course plots of I_Ba-step_ (open circle) and I_Ba-ramp_ (open square) for the entire experiment. The boxed region was expanded and shown in **(G)**. **G)** Time course plots of I_Ba-step_ (*top*) and I_Ba-ramp_ (*bottom*) from traces 60 to 200 and bath temperature (gray dotted line). Both I_Ba-step_ and I_Ba-ramp_ were corrected for rundown, using 0.00741 nA/trace and 0.00538 nA/trace, respectively. **H)** R_input_ (*top*) and I_-80 mV_ (*bottom*). **I)** Rundown-corrected I_Ba-step_ (*left*) or I_Ba-ramp_ (*right*) vs. bath temperature. Fitting I_Ba-step_ vs. temperature plot with a linear function yielded a slope of -0.147 nA/°C and a Y-intercept of 2.947 nA (r = -0.88). Rundown-corrected I_Ba-ramp_ vs. temperature plot (r = -0.36). **J)** Calculated and normalized I_Ba-step_ at 37°C (black circle) and 34°C (gray circle).

During near PT recordings, the step current sometimes showed amplitude fluctuations that occurred without changes in the passive membrane properties (i.e., R_input_ or I_-80 mV_; S3 Fig). As these amplitude fluctuations were not observed with RT recordings, one hypothesis is that they are associated with temperature fluctuations during the near PT recordings. Therefore, temperature sensitivity of the Ca_V_1.2 current was examined. These experiments were conducted using setups with the TC-344C controller, as amplitude fluctuations were more common when recorded using these setups, and the thermistor measuring the bath temperature was positioned as close to the recorded cell as possible. After the current reached a quasi-steady state level at near PT, temperature control of the aluminum platform was turned off to allow graded bath temperature drop at the recording chamber, and then back on to elevate the bath temperature. Fig 2A shows the time course plots of I_Ca-step_ and I_Ca-ramp_ from a representative cell. The boxed regions are expanded and shown in Fig 2B. I_Ca-step_ decreased and increased as the bath temperature lowered and elevated, respectively (*top*; note that Ca^2+^ current amplitudes are expressed as negative values), while I_Ca-ramp_ appeared to show the opposite pattern (*bottom*). No change in R_input_ or I_-80 mV_ was observed (Fig 2C). The left panel of Fig 2D shows the plot of I_Ca-step_ vs. temperature for this cell, demonstrating a strong linear relationship (r = -0.94). In contrast, no relationship was observed between I_Ca-ramp_ and temperature (r = 0.38; Fig 2D, *right*). Temperature sensitivity of the Ca^2+^ current was confirmed in 5 more cells (S4A–S4E Fig). In all cells recorded, I_Ca-step_ showed strong linear relationship with temperature (r = -0.77 to -0.94). The I_Ca-ramp_-temperature relationship was inconsistent: 4 cells showed no relationship (Fig 2D, *right*; S4A, S4B and S4D Fig., *right*), and 2 showed linear relationship (r = -0.73 to -0.88). Fig 2E shows the calculated and normalized I_Ca-step_ at 37°C and 34°C. Depending on the cell, 3°C of temperature drop reduced I_Ca-step_ amplitude by 12% to 34%. This magnitude of temperature fluctuation was routinely observed during near PT experiments for setups with TC-344 controller.

Temperature sensitivity of the Ba^2+^ current was examined as well. Fig 2F shows the time course plots of I_Ba-step_ and I_Ba-ramp_ from a representative cell, and the boxed region was expanded and shown in Fig 2G. I_Ba-step_ changed in the same direction as the bath temperature (*top*), and I_Ba-ramp_ seemed to not respond to temperature changes (*bottom*). R_input_ and I_-80 mV_ remained stable during temperature manipulations (Fig 2H). The left panel of Fig 2I shows the plot of I_Ba-step_ vs. temperature, demonstrating a clear linear relationship (r = -0.88). The plot of I_Ba-ramp_ vs. temperature, on the other hand, did not reveal any relationship (r = -0.36; Fig 2I, *right*). Temperature-dependent effect on the Ba^2+^ was confirmed in 4 more cells (S4F–S4I Fig). In all cells recorded, I_Ba-step_ showed strong linear relationship with temperature (r = -0.87 to -0.93). The data for I_Ba-ramp_ and temperature were inconsistent: 3 cell showed no-to-weak relationship (Fig 2I, *right*; S4F and S4G Fig, *right*), and 2 showed strong relationship (r = -0.80 and -0.88). Fig 2J shows the calculated and normalized I_Ba-step_ at 37°C and 34°C. Depending on the cell, 3°C temperature drop reduced I_Ba-step_ by 18% to 28%.

The step current in the present study is very temperature-sensitive regardless of which charge carrier was used. These results are consistent with prior studies conducted on cloned Ca_V_1.2 channels comprised of α_1c_, α_2_/δ_a_, and β_1b_ (or β_2c_) expressed in xenopus oocytes [23] and L-type Ca^2+^ channels in native ventricular myocytes [24, 25] that also showed high temperature sensitivity. Increasing PKA-mediated phosphorylation reduced temperature sensitivity of some Ca^2+^ channel gating parameters, suggesting that the high temperature sensitivity may be due to channels in the unphosphorylated state [25]. Temperature sensitivity of the step current adds an additional challenge to conducting pharmacology experiments at near PT, since several degrees of temperature fluctuations can easily be produced by a slowing of the flow rate due to the presence of bubbles in the perfusion line and/or reduced fluid level in the reservoirs of the gravity-fed perfusion system. Temperature sensitivity of the Ca_V_1.2 current is thus a source of variability for pharmacology data generated within and across laboratories.

### Consequence of R_s_ compensation on Ca_V_1.2 current at near PT

**Fig 3:**
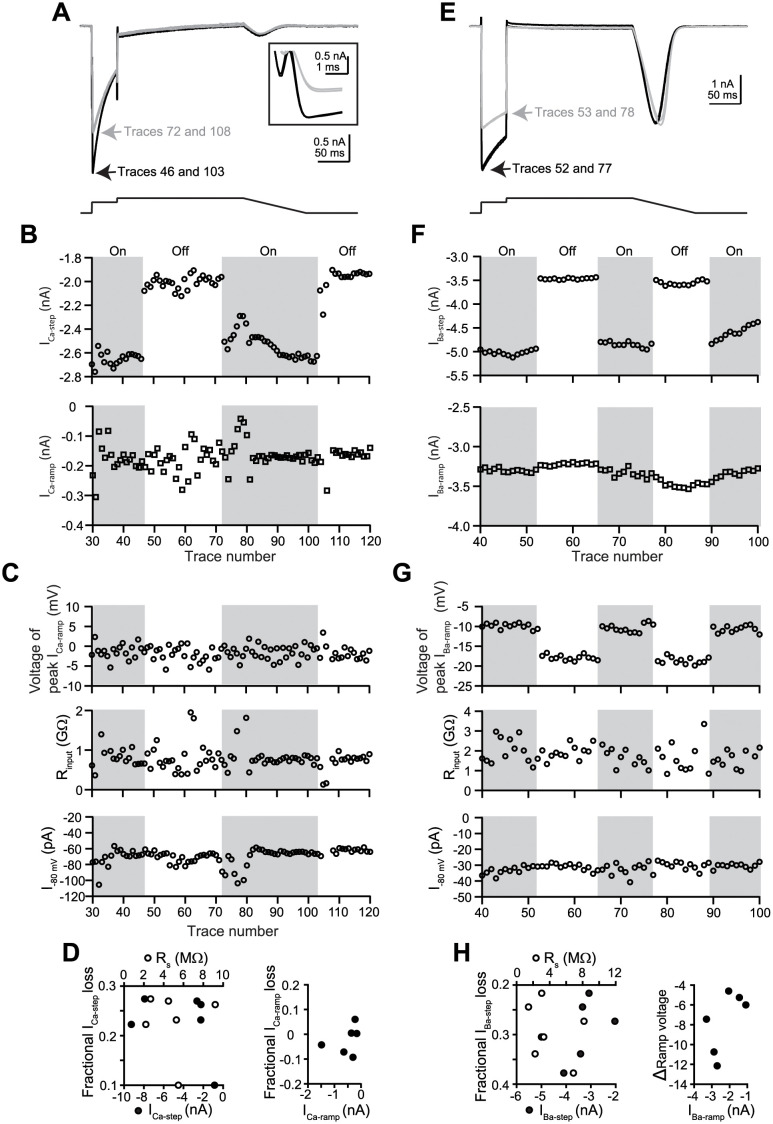
Effects of R_s_ compensation on Ca^2+^ and Ba^2+^ currents. Data shown in panels **(A)** through **(D)** were obtained in external Ca^2+^. Cell ID was 21318008 for panels **(A)** through **(D)**. **A)** *Top*, representative Ca^2+^ current traces obtained from a cell, with (black traces) and without R_s_ compensation (gray traces). *Bottom*, the voltage protocol used. **B)** Time course plots of I_Ca-step_ (*top*) and I_Ca-ramp_ (*bottom*), focusing on traces 30 to 120 for which R_s_ compensation was turned on and off. No rundown correction was made for these plots. **C**) Time course plots of ramp voltage at which I_Ca-ramp_ reached peak amplitude (*top*), R_input_ (*middle*), and I_-80 mV_ (*bottom*) for traces 30 to 120. **D**) *Left*, fractional I_Ca-step_ loss due to not compensating for R_s_, calculated as the ratio of I_Ca-step_ without R_s_ compensation vs. I_Ca-step_ with 80% R_s_ compensation, and plotted against I_Ca-step_ obtained with R_s_ compensation (lower x-axis) or the amount of R_s_ compensated (upper x-axis). *Right*, fractional I_Ca-ramp_ loss due to not compensating for R_s_, plotted against I_Ca-ramp_ with R_s_ compensation. Data shown in panels **(E)** through **(H)** were obtained in external Ba^2+^. Cell ID was 2021_06_25_0004 for panels (**E**) through (**I**). **E**) Representative Ba^2+^ current traces obtained from a cell, with (black traces) and without R_s_ compensation (gray traces). **F)** Time course plots of I_Ba-step_ (*top*) and I_Ba-ramp_ (*bottom*), focusing on traces 40 to 100 during which R_s_ compensation was turned on and off. No rundown correction was made for these plots. **G**) Time course plots of ramp voltage (*top*), R_input_ (*middle*), and I_-80 mV_ (*bottom*) for traces 40 to 100. **H**) *Left*, fractional I_Ba-step_ loss due to not compensating for R_s_, plotted against I_Ba-step_ obtained with R_s_ compensation (lower x-axis) or the amount of R_s_ compensated (upper x-axis). *Right*, delta (Δ) ramp voltage shift for I_Ba-ramp_ due to not compensating for R_s_, plotted against I_Ba-ramp_ with R_s_ compensation.

The cells used to generate Fig 1 were used to estimate the activation kinetics and amplitude of Ca_V_1.2 current at near PT. I_Ca-step_ reached peak 1.84 ± 0.17 ms following the voltage jump to 0 mV (range: 1.34 to 3.39 ms; n = 12, using cells with clear separation between capacitive transient and the step current), and was -2210.1 ± 178.3 pA in amplitude (range: -1376.7 to -3362.9 pA, based the average of 91^st^ to 100^th^ traces recorded in the control solution). I_Ca-ramp_ reached peak at 0.6 ± 0.6 mV during the repolarizing ramp (range: -4.0 to 3.6 mV; n = 16), and was -814.0 ± 108.4 pA in amplitude (range: -320.2 to -1546.3 pA). The fast kinetics and relatively large amplitudes of the step current prompted for a set of experiments to assess the impact of R_s_ compensation in these recordings. During these experiments, R_s_ was measured and compensated at 80% before the start of recording. After Ca_V_1.2 current reached a quasi-steady state level in the control solution, R_s_ compensation was turned “off” and “on” as the recording continued. Fig 3A and 3B show representative current traces and time course plots of I_Ca-step_ and I_Ca-ramp_ from one cell obtained with and without R_s_ compensation. The total initial R_s_ of this cell was 5.6 MΩ. With R_s_ compensation, I_Ca-step_ was ~600 pA larger than without R_s_ compensation (Fig 3B, *top*). In contrast, no difference was observed in I_Ca-ramp_ amplitude (*bottom*) or the ramp voltage at which I_Ca-ramp_ reached peak (Fig 3C, *top*). R_input_ and I_-80 mV_ remained stable (Fig 3C, *middle and bottom*). The impact of R_s_ compensation was confirmed in 5 additional cells. The left panel of Fig 3D shows the fractional I_Ca-step_ loss without R_s_ compensation for all 6 cells, plotted against either I_Ca-step_ with R_s_ compensation (solid symbols) or the amount of R_s_ that was compensated (or 80% total R_s_; open symbols). No relationship was evident amongst these parameters, as expected since voltage loss through R_s_ (hence fractional current loss) is a product of both the current amplitude and R_s_. For these cells, voltage loss through R_s_ at I_Ca-step_ ranged from 4.7 to 21.3 mV, calculated by using Ohm’s law. The right panel of Fig 3D shows the fractional I_Ca-ramp_ loss due to not compensating for R_s_, plotted as a function of I_Ca-ramp_ with R_s_ compensation. Collectively, no loss was observed (average: -0.02 ± 0.02).

The kinetics and amplitude of Ba^2+^ current at near PT was also quantified. I_Ba-step_ reached peak 2.91 ± 0.25 ms following the voltage jump to 0 mV (range: 1.64 to 4.23 ms; n = 11), and was -4147.6 ± 813.1 pA in amplitude (range: -1420.0 to -8395.0 pA). I_Ba-ramp_ reached peak at -7.7 ± 0.7 mV (range: -2.5 to -10.5 mV; n = 11) and was -2077.7 ± 309.5 pA in amplitude (range: -555.2 to -3506.4 pA). Fig 3E showed representative Ba^2+^ current traces obtained from a cell, with and without R_s_ compensation. The total R_s_ for this cell was 4.1 MΩ. Similar to Ca^2+^ current recordings, R_s_ compensation affected the amplitude of I_Ba-step_ (by ~1500 pA; Fig 3F, *top*) but not the amplitude of I_Ba-ramp_ (Fig 3F, *bottom*). However, the ramp voltage at which I_Ba-ramp_ peaked was consistently shifted rightward when R_s_ was not compensated, toward the more hyperpolarized potential (Fig 3G, *top*). No change in R_input_ or I_-80 mV_ was observed (Fig 3G, *middle and bottom*). The impact of R_s_ compensation on the Ba^2+^ current was verified in 5 more cells. The left panel of Fig 3H shows the fractional I_Ba-step_ loss due to not compensating for R_s_, plotted against I_Ba-step_ amplitude with R_s_ compensation (solid symbols) or the amount of R_s_ compensated (open symbols). No relationship was evident amongst these parameters. Voltage loss through R_s_ at I_Ba-step_ ranged from 4.8 to 28.2 mV. The right panel of Fig 3H summarizes the hyperpolarizing shift of the ramp voltage for I_Ba-ramp_ without R_s_ compensation. On average, the shift was -7.7 ± 1.3 mV (range: -4.6 to -12.1 mV) and was not accompanied by I_Ba-ramp_ amplitude change (fractional I_Ba-ramp_ loss: 0.03 ± 0.00). Using normalized I-V relation generated from 15 cells, the reversal potential of Ba^2+^ current was estimated to be +35 mV (S5A Fig). The increase in Ba^2+^ driving force due to the hyperpolarizing shift in the ramp voltage offers an explanation as to why I_Ba-ramp_ amplitude was unaltered by not compensating for R_s_, since this could compensate for the fewer number of Ca_V_1.2 channels that would be open due to not clamping the membrane potential at the expected levels.

These results demonstrate the impact of R_s_ compensation when recording fast and large Ca^2+^ and Ba^2+^ currents at near PT. While R_s_ compensation is a good practice for voltage clamp experiments, can R_s_ affect pharmacology results? A recent study that modeled drug block of Na^+^ channels indicates so, with the degree of rightward shift of the concentration-inhibition graphs dependent on the magnitudes of R_s_ and Na^+^ current [26]. R_s_ compensation is a practice that also differed between Crumb et al., 2016 (compensated) [5] and Li et al., 2018 (did not compensate) [6] (Table 1). Therefore, the decision to apply R_s_ compensation or not during voltage clamp experiments to characterize drug effects may also be a contributing factor inter-laboratory data variability.

### Effects of recording temperature on verapamil and methadone inhibition of Ca_V_1.2 current

**Fig 4:**
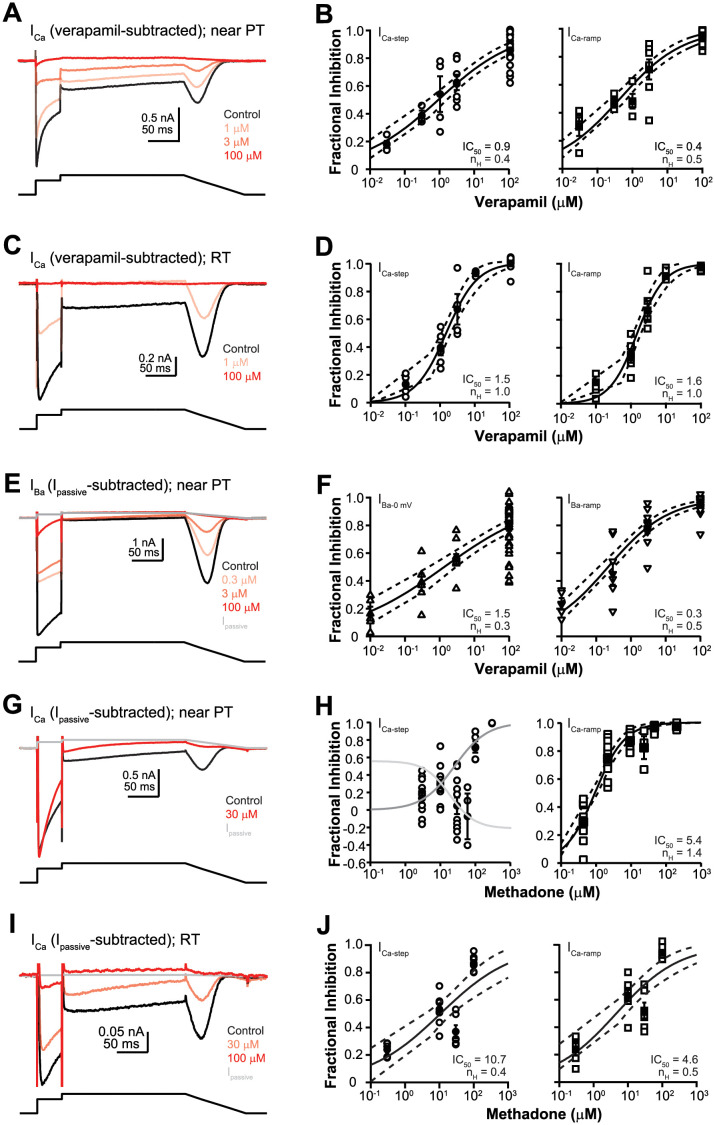
Ca_V_1.2 channel block by verapamil and methadone at near PT and RT. **A)** Example current traces recorded from one cell (cell ID: 19820002) in external Ca^2+^ at near PT. The illustrated traces were obtained in the control solution (black; trace 99), and following application of 1 μM (light red, trace 160), 3 μM (medium red, trace 209), and 100 μM verapamil (dark red; trace 250). This cell exhibited an outward current that was unmasked by 100 μM verapamil. Thus, Ca_V_1.2 current was isolated using the verapamil-subtraction method. **B)** Concentration-inhibition plots for I_Ca-step_ (*left*) and I_Ca-ramp_ (*right*) at near PT. Data points from individual cells were shown in open symbols (I_Ca-step_, circle; I_Ca-ramp_, square). Group averages (± sem) for different concentrations were shown in solid symbols plus error bars. The solid sigmoidal curve indicates the fit using the Hill equation; the dashed curves, upper and lower limit of the 95% CI of the fit. **C)** Example current traces recorded from one cell (cell ID: 19d31003) in external Ca^2+^ at RT. The illustrated traces were obtained in the control solution (black; trace 54), and following application of 1 μM (light red, trace 110) then 100 μM verapamil (dark red; trace 155). This cell also exhibited an outward current that was unmasked by 100 μM verapamil. Thus, Ca_V_1.2 current was isolated by using verapamil-subtraction method. **D)** Concentration-inhibition plots for I_Ca-step_ (*left*) and I_Ca-ramp_ (*right*) at RT. **E)** Example current traces recorded from one cell (cell ID: 19d04004) in external Ba^2+^ at near PT. The illustrated traces were obtained in the control solution (black; trace 82), and following application of 0.3 μM (light red, trace 171), 3 μM (medium red, trace 228), and 100 μM verapamil (dark red; trace 286). I_passive_ (gray) was calculated based on R_input_ derived from trace 286. This cell had little to no outward current in the presence of verapamil, and Ca_V_1.2 current was isolated using I_passive_-subtraction method. **F)** Concentration-inhibition plots for I_Ba-step_ (*left*) and I_Ba-ramp_ (*right*) at RT. **G)** Example current traces recorded from one cell (cell ID: 19508007) in external Ca^2+^ at near PT. The illustrated traces were obtained in the control solution (black; trace 115), and following application of 30 μM methadone (red, trace 196). I_passive_ (gray) was calculated based on R_input_ derived from trace 196. Note that in the presence of methadone, I_Ca-step_ amplitude was slightly increased while its decay was accelerated. **H)** Concentration-inhibition plots for I_Ca-step_ (*left*) and I_Ca-ramp_ (*right*) at near PT. For visual presentation only, individual data points at 10, 30, and 60 μM reflecting methadone’s facilitatory effect were fit with the Hill equation (light gray curve). To illustrate the inhibitory effect on I_Ca-step_, individual data points excluding 30 and 60 μM methadone were fit with the Hill equation (dark gray curve). **I)** Example traces of current recorded from one cell (cell ID: NTCell_2019_08_29_0013) in external Ca^2+^ at RT. The illustrated traces were obtained in the control solution (black; trace 100), and following application of 30 (medium red, trace 159) and 100 μM methadone (red, trace 250). I_passive_ (gray) was calculated based on R_input_ derived from trace 250. **J)** Concentration-inhibition plots of methadone’s effects at RT.

The preceding sections focused on the Ca^2+^ and Ba^2+^ current characteristics at near PT. The next two sections focus on the impact of specific experimental factors on Ca_V_1.2 channel pharmacology. The effect of recording temperature on Ca_V_1.2 channel block was examined for verapamil and methadone. Fig 4A and 4C show representative recordings of Ca^2+^ current obtained in the control solution and following verapamil application at near PT and RT, respectively. Fig 4B and 4D show concentration-inhibition plots of verapamil for I_Ca-step_ and I_Ca-ramp_ at these temperatures. At near PT, the IC_50_ of verapamil inhibition of I_Ca-step_ was 0.9 μM. This is 2.5X the IC_50_ of I_Ca-ramp_, which was 0.4 μM. The more potent inhibition at I_Ca-ramp_ suggests continued block development throughout the 0 and the +30 mV steps that led to fewer channels available to reactivate during the repolarizing ramp. Since Cav1.2 channels enter inactivation following activation by the 0 mV step, a more potent inhibition of the ramp current relative to the step current using the present voltage protocol suggests inactivated state block in addition to the open state block (inferred by inhibition of the step current). At RT, the IC_50_s of verapamil for I_Ca-step_ and I_Ca-ramp_ were 1.5 and 1.6 μM, respectively. The equally potent inhibition of the step and the ramp current at RT suggests a preference for open channel block. A surprisingly finding was that the slope of the concentration-inhibition relationship was temperature-dependent. At near PT, the n_H_ values for I_Ca-step_ and I_Ca-ramp_ were 0.4 and 0.5, respectively. At RT, these values were 1 (Fig 4B and 4D). Assuming a single binding site, a n_H_ value of 1 as observed at RT indicates a tight coupling between verapamil binding and block of Ca^2+^ permeation, consistent with a direct pore blocking mechanism. On the other hand, the n_H_ values of 0.4 and 0.5 as observed at near PT suggests a weaker coupling between verapamil binding and effect at the channel pore. This may occur if verapamil inhibition at near PT involves allosteric mechanisms (i.e., drug binding does not directly block Ca^2+^ permeation, but rather induces channel closure or locking in the inactivated state). Verapamil inhibition of the Ba^2+^ current was also studied at near PT (Fig 4E and 4F). Relative to the drug effect on the Ca^2+^ current at near PT, the difference between the IC_50_s of the step and the ramp current was more pronounced in external Ba^2+^. The IC_50_ at I_Ba-step_ was 1.5 μM, 5X the IC_50_ of I_Ba-ramp_ which was 0.3 μM. The faster block development at the 0 and +30 mV step in external Ba^2+^ could be due to verapamil interactions with either open and/or inactivated channel state, since more channels remained open at these voltages due to removal of Ca^2+^-dependent inactivation. The n_H_ values were low also, 0.3 and 0.5, respectively, similar to the drug effect on the Ca^2+^ current at near PT and inconsistent with a direct pore block mechanism.

The consequence of recording temperature was also assessed for methadone on the Ca^2+^ current. Fig 4G through Fig 4J show representative cells and corresponding concentration-inhibition plots at near PT and RT, respectively. At near PT, methadone exhibited complex effects on I_Ca-step_. Collectively, facilitation was seen at 30 and 60 μM, and inhibition was seen at higher concentrations. The facilitatory effect was characterized by an increase in the peak amplitude, accelerated current decay during the 0 mV step (Fig 4G), and was specific for I_Ca-step_ (Fig 4H). The latter point is clearly illustrated in Fig 4G: as I_Ca-step_ showed a slight increase in 30 μM methadone, I_Ca-ramp_ was nearly completely inhibited (Fig 4G). Given the complex effects at I_Ca-step_, methadone’s IC_50_ was estimated for I_Ca-ramp_ only, which was 5.4 μM with n_H_ of 1.4. At RT, only inhibitory effect was observed. The IC_50_ for I_Ca-step_ at RT was 10.7 μM with n_H_ of 0.4, and that for I_Ca-ramp_ was 4.6 μM with n_H_ of 0.5. These results suggest that methadone blocks Ca_V_1.2 channels in both open and inactivated states at RT. This conclusion is consistent with a previous study that reported IC_50_s of 26.6 μM for tonic or open channel block, and 7.7 μM for phasic or inactivated channel block, respectively, for methadone at ambient temperature [27]. In summary, methadone has dual effects at PT but not RT. Like verapamil, the n_H_ value for methadone’s inhibitory effect is temperature-dependent. Dual effects of drugs on L-type Ca^2+^ channels in native myocytes that depend on the membrane potential have also been reported for dihydropyridine (+)-202-791 [28] and nitrendipine [29]. For nitrendipine, the concentration-facilitation plot clearly illustrated an inflection point, suggesting the existence of two binding sites with different affinities on the Ca^2+^ channels [29]. Methadone used in the present study is a racemic mixture. Another study has reported that the R- and S-enantiomers of the cyclin-dependent kinase inhibitor roscovitine bind to different sites on Ca_V_1.2 channels to affect activation and inactivation separately [30]. It is tempting to reconcile the present results by proposing distinct binding sites for methadone on Ca_V_1.2 channels that are accessible at near PT but not RT. Follow-up studies are required to test this possibility.

A few drugs have been shown to exhibit temperature-dependent block on Ca_V_1.2 channels in overexpression cells [20] or L-type Ca^2+^ channels in native myocytes [21]. One study showed that nitrendipine and diltiazem inhibited Ca^2+^ current mediated by Ca_V_1.2, β_1_, α_2_/δ, and γ subunits more potently at RT than at 33°C [7]. Another study reported that increasing the recording temperature from 22°C to 37°C increased the block potencies of flavoxate and nifedipine on L-type Ca^2+^ channels by 2.2X and 7X, respectively [8]. The direction and magnitude of potency shift due to temperature is thus drug-specific. Results of verapamil and methadone from the present study further extend those in the literature, demonstrating that recording temperature is an experimental factor that impacts Ca_V_1.2 pharmacology.

### Comparisons of drug effects on the Ca^2+^ and Ba^2+^ currents at near PT

**Fig 5:**
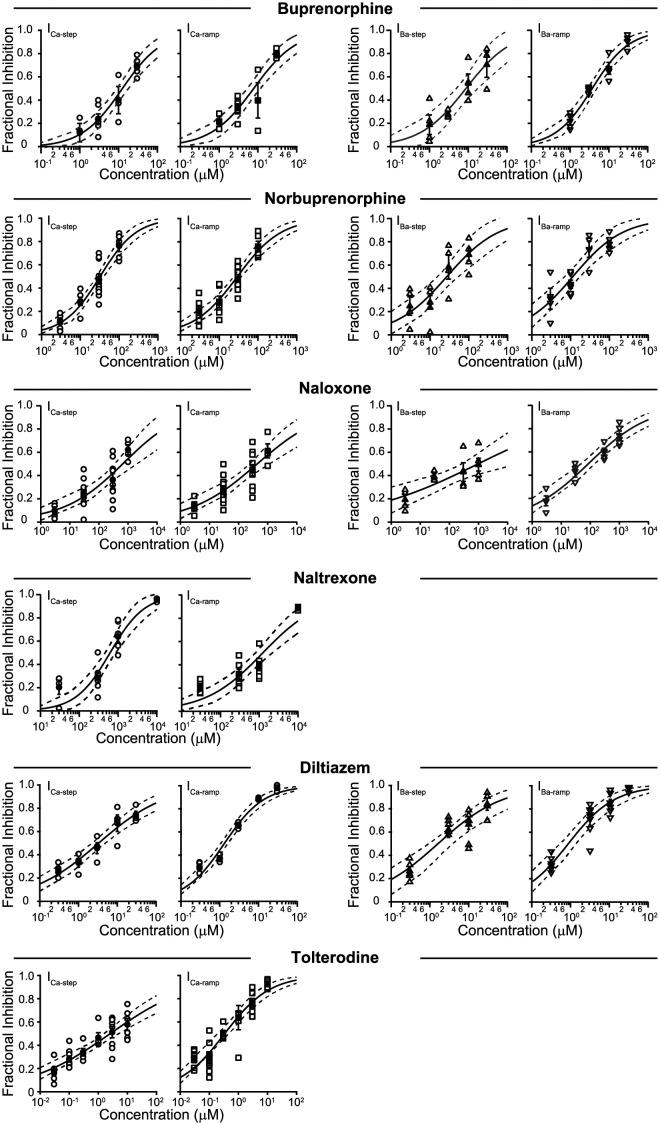
Concentration-inhibition plots for Ca_V_1.2 channel block by buprenorphine, norbuprenorphine, naloxone, naltrexone, diltiazem, and tolterodine at near PT. Data for I_Ca-step_ are shown in circles; I_Ca-ramp_, squares; I_Ba-step_, upright triangles; I_Ba-ramp_, inverted triangles. Open symbols reflect individual data points; filled symbols plus error bars, mean ± sem. The solid sigmoidal curve indicates the fit with the Hill equation; the dashed curves, upper and lower limit of the 95% CI of the fit.

**Fig 6:**
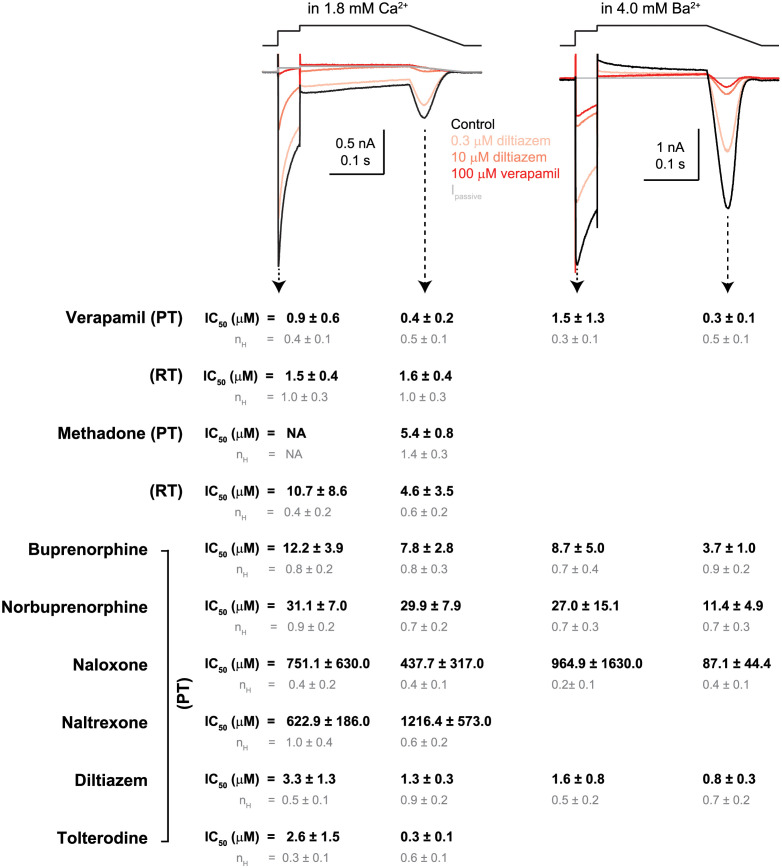
Summary of IC_50_ and n_H_ values. *Top*. The voltage protocol used and example traces recorded at near PT from two cells, one recorded in external Ca^2+^ (*left*; cell ID: 21515003c) and the other in external Ba^2+^ (*right*; cell ID: 21617012). For the cell on the left, the illustrated traces were obtained in the control solution (black; trace 206), 0.3 μM diltiazem (light red; trace 278), 10 μM diltiazem (medium red; trace 339), and 100 μM verapamil solutions (dark red; trace 397). For the cell on the right, the illustrated traces were obtained in the control solution (black; trace 50), 0.3 μM diltiazem (light red; trace 130), 10 μM diltiazem (medium red; trace 240), and 100 μM verapamil solutions (dark red; trace 286). I_passive_ traces, shown in gray, were calculated based on R_input_ derived from the verapamil traces shown. The voltage protocol was overlaid on top of the current traces. Note that for the cell on the right, Ba^2+^ current isolation was done using I_passive_ subtraction, as the outward current seen in control solution was no longer apparent in diltiazem and verapamil solutions. *Bottom*, summary IC_50_ and n_H_ values, (mean ± 95% CI) obtained at the current regions indicated by the dotted arrows.

**Fig 7:**
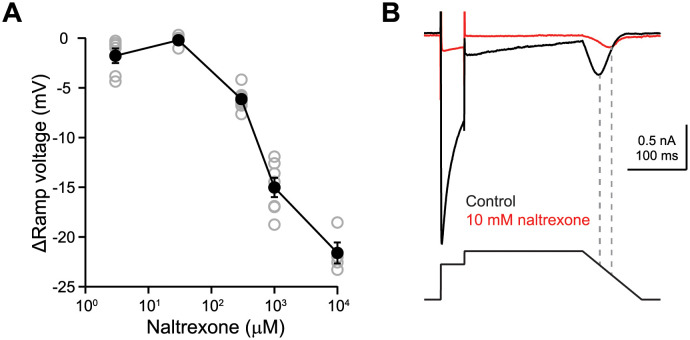
Concentration-dependent shift of voltage at which I_Ca-ramp_ peaked. **A)** Changes in the ramp voltage vs. naltrexone concentrations. Gray open symbols indicated data points from individual cells; black filled symbols plus error bars indicate mean ± sem. **B)** Representative current traces obtained from 1 cell (cell ID: 20109006) in external Ca^2+^ at near PT. The 90^th^ trace (black) was the last trace obtained in the control solution; the 140^th^ trace (red) was obtained following 10 mM naltrexone application. The voltage protocol was shown below the current traces. In this cell, the ramp voltage that I_Ca-ramp_ reached peak shifted from –2 mV in the control solution to -18 mV following naltrexone application. For the naltrexone data set, the ramp voltage at which peak I_Ca-ramp_ occurred in the control solution was 0.15 ± 0.35 mV (n = 21), consistent with the cells used to generate Fig 1C, 1D and 1G.

The effects of buprenorphine, norbuprenorphine, naloxone, and diltiazem were studied at near PT on the Ca^2+^ and Ba^2+^ currents; of naltrexone and tolterodine, on the Ca^2+^ current alone. Fig 5 shows the concentration-inhibition plots for these drugs. Fig 6 summarizes the IC_50_s and n_H_ values for all drugs studied. For near PT recordings, verapamil, buprenorphine, naloxone, diltiazem, and tolterodine inhibited I_Ca-ramp_ more potently than I_Ca-step_, suggesting that these drugs all have affinity for open and inactivated channels. Tolterodine showed the largest I_Ca-ramp_ vs. I_Ca-step_ IC_50_ difference, by a factor of 8.7, suggesting a stronger preference for the inactivated state comparing with other drugs. For norbuprenorphine, there was no difference in the IC_50_s for I_Ca-step_ and I_Ca-ramp_, suggesting a preference for open channel block when Ca^2+^ was used as the charge carrier. Naltrexone was the only drug tested that showed higher IC_50_ for I_Ca-ramp_ than for I_Ca-step_. This drug produced a dramatic concentration-dependent hyperpolarizing shift in the ramp voltage (Fig 7A), by -22 mV at 10 mM (Fig 7B), demonstrating an effect on voltage-dependence of channel gating. Using I-V generated from 14 cells, the reversal potential for Ca^2+^ current under the current experimental condition was estimated to be +46 mV (S5B Fig). The lower fractional inhibition of I_Ca-ramp_ relative to I_Ca-step_ thus may not indicate drug unbinding during the +30 mV step. Instead, the increased driving force through channels that are available at more hyperpolarized membrane voltages in the presence of naltrexone may also be an explanation of the higher IC_50_ for I_Ca-ramp_ than for I_Ca-step_. S6 and S7 Figs provide time course plots of individual cells tested with select drugs in Ca^2+^ and Ba^2+^, respectively.

These results demonstrate that even when drug effects were analyzed within the same cell using the same traces, different drug potencies could be obtained depending on which current region was analyzed. Similar analyses have been performed for (-)-menthol and nimodipine [15]. In rabbit ventricular myocytes, these drugs inhibited the peak Ca^2+^ current evoked by a step depolarization less potently than the late Ca^2+^ current that remained at the end of the step depolarization, with nimodipine showing 13.1X difference in the IC_50_s. The difference in IC_50_s obtained for the step and the ramp current are compatible with literature findings of drugs exhibiting state- and/or use-dependent block of Ca_V_1.2 channels in overexpression cell lines and L-type Ca^2+^ channels in native myocytes. Nitrendipine [13, 17], nisoldipine, nicardipine [17], nifedipine, verapamil [16], and mibefradil [14] all showed more block when the cells were held at depolarized membrane potential than when cells were held at hyperpolarized membrane potential, suggesting preferential block of channels in the inactivated state. Verapamil and diltiazem showed block increasing at higher stimulation frequencies and higher depolarizations, suggesting a preference for the open and inactivated state over closed state [9–11, 31].

Figs 5 and 6 also show data summarizing inhibition of the Ba^2+^ current by verapamil, buprenorphine, norbuprenorphine, naloxone, and diltiazem. All drugs showed greater inhibition of I_Ba-ramp_ than I_Ba-step_ (Figs 5 and 6) The largest difference was seen for verapamil, for which IC_50_s between the step and the ramp current differed by a factor of 5. In external Ba^2+^, the differences between the IC_50_s of the step and the ramp current were more pronounced than in external Ca^2+^ for verapamil, buprenorphine, norbuprenorpine, and naloxone. Even norbuprenorphine, which showed no difference between the IC_50_s of I_Ca-step_ and I_Ca-ramp_, showed a difference of 2.4X between I_Ba-step_ and I_Ba-ramp_. These results thus demonstrate that state-dependent interactions between drugs and Ca_V_1.2 channels, as well as relative preference for different channel states, are dependent on the charge carrier used. Generalizations regarding drug-Ca_V_1.2 channel interactions based on different studies should thus take the charge carrier used into consideration.

Comparisons of the IC_50_s on the same current region obtained in external Ba^2+^ and Ca^2+^ showed a small impact on the step current. Based on point estimate comparison, diltiazem showed the biggest difference, with an IC_50_ at I_Ca-step_ 2.1X higher than that at I_Ba-step_. Likewise, the impact of charge carrier on the ramp current and the direction of shift were also drug-specific. Verapamil showed no difference, while naloxone showed a 5X difference, with I_Ba-ramp_ being more sensitive to block than I_Ca-ramp_. Results of the present study thus add to those in the literature demonstrating an impact of charge carrier on drug inhibition of Ca^2+^ and Ba^2+^ currents. In ventricular myocytes, diltiazem [11], D600 (a methoxy derivative of verapamil [11]), and verapamil [12] were less effective in inhibiting Ba^2+^ current than Ca^2+^ current through Ca^2+^ channels [11]. Similar findings were reported for verapamil [10] and diltiazem [9] studied using Ca_V_1.2 channels with β_1b_ and α_2_δ subunits using overexpression cells.

Data in Figs 5 and 6 also show that the n_H_ values were quite variable amongst the drugs, ranging from very low (n_H_ = 0.2 for naloxone on I_Ba-step_) to quite steep (n_H_ = 1.4 for methadone on I_Ca-ramp_). Shallow slopes of the concentration-inhibition relationship may be reflective of technical challenges in the experiments. These challenges include current rundown that can inflate estimation of fractional inhibition at low drug concentration, and insolubility at high drug concentration that leads to fewer than expected free drug molecules to block ion channels. While these possibilities cannot be ruled out for tolterodine, they cannot explain data of other drugs. For verapamil and methadone, changing the recording temperature greatly altered the steepness of the concentration-inhibition curves, with n_H_ values at one temperature being 0.4 to 0.6 and at the other temperature being 1 and above. For naltrexone and diltiazem, n_H_ values differ between the step and the ramp current, with that for one current approaching 1 while the other remaining at or below 0.6. For naloxone and naltrexone, this laboratory has previous tested similar concentrations on Na_V_1.5 channels and obtained n_H_ values equal to or greater than 0.8 on the peak and the late current [18]. Therefore, the simplest explanation is that for these drugs, the shallowness of the concentration-inhibition relationship reflects more complex drug-Ca_V_1.2 channel interactions that cannot be readily explained by 1:1 drug-receptor binding scheme that leads to immediate current inhibition. It is difficult to relate these n_H_ results to existing literature, since n_H_ values for concentration-inhibition plots are often not reported. In a study that assessed the structural basis of diltiazem block of voltage-gated Ca^2+^ channels, the resting state block was found to have an IC_50_ of 41 μM and a much steeper slope for the concentration-inhibition relationship than use-dependent block, which had a shallower slope but more potent block with an IC_50_ of 10.4 μM [32]. These results therefore demonstrate that n_H_ values for diltiazem are state-dependent, consistent with the present findings. Based on X-ray crystallographic analysis, the study showed that diltiazem has two distinct binding poses with the Ca^2+^ channels: upon entering the channel pore, this drug forms a loose channel-blocking complex that appears to be a low affinity binding mode, and then rearranges within the channel to a tighter binding, more stably blocking complex with diltiazem projecting into the selectivity filter from the central cavity upon voltage-dependent inactivation [32]. Importantly, diltiazem binding also allosterically modulates Ca^2+^ binding in the selectivity filter, suggesting this mechanism may also contribute to reduction of current. Therefore, for a “pore blocker” like diltiazem, the different binding poses associated with different channel states may explain different n_H_ values.

Table 2 summarizes the IC_50_ values reported in the literature for methadone, diltiazem, verapamil, and tolterodine as well as experimental protocol used. A wide range of IC_50_s were reported, with the max-to-min ratios for diltiazem being 397X (0.63 μM vs. 250 μM) and for verapamil 294X (0.16 μM vs. 47 μM). The only study that reported Ca_V_1.2 channel block by buprenorphine, norbuprenorphine, naltrexone, and naloxone was by this laboratory [18]. In Tran et al., 2020, the IC_50_ values of drug block on the ramp current measured using Ca^2+^ as the charge carrier was derived from the same cells used in the present study.

**Table 2:** Comparisons of IC_50_ values for Ca_V_1.2 channel block for methadone, diltiazem, and verapamil generated by different experimental protocols.

Drug	Cell type	Subunits	Platform	Recording temperature (ºC)	Charge carrier (mM)	Voltage protocol	IC_50_ and n_H_ (± SE)	Reference
Methadone	CHO	hCa_V_1.2, β_2_, α_2_δ	Automated (PatchXpress 7000A)	Ambient	NA	2-pulse protocol: a 500 ms step from -80 to +10 mV, followed by repolarization to -80 mV for 500 ms, followed by a 200 ms step to +10 mV; the pulse pattern was repeated at 0.1 Hz	26.3 μM (tonic block) 7.7 μM (phasic block)	[27]
Diltiazem	tsA-201 cells	Ca_V_1.2, α_2_δ, β_1b_	Manual	RT	10 Ba^2+^	100 ms pulses from -60 mV to +10 mV at 0.05 Hz	65 ± 13 μM	[9]
	tsA-201 cells	Ca_V_1.2, α_2_δ, β_2a_	Manual	22–25ºC	20 Ba^2+^	100 ms pulses from -80 mV to +20 mV at 0.2 Hz	85 ± 9 μM	[38]
	HEK293	Ca_V_1.2, α_2_δ, β_2a_ along with K_ir_2.3	Automated (PatchXpress 7000A) and manual	Automated 22–26ºC; manual 20–25ºC	30 Ba^2+^	100 ms step from -60 to +20 mV at 0.05 Hz	36 μM (automated) 44 μM (manual)	[39]
	Guinea pig ventricular myocytes	L-type Ca^2+^ current	Manual	20–25ºC	10 Ca^2+^	200 ms step from -70 to +10 mV at 0.2 Hz	30 μM	[39]
	Guinea pig ventricular myocytes	L-type Ca^2+^ current	Manual	RT, defined as 22–25ºC in [40]	2 Ca^2+^	200 ms step from -50 to +10 mV at 0.2 Hz	0.63 ± 0.05 μM; n_H_ = 0.77	[41]
	CHO	hCa_V_1.2, β_2_, α_2_δ	Automated (QPatch and PatchXpress)	Ambient	1.8 Ca^2+^	150 ms pulse from -40 mV to 0 mV every 5 s	0.76 ± 0.08 μM; n_H_ = 1.14 ± 0.15	[33]
	*Trichopulsia ni* cells (insect)	Ca_V_Ab (voltage-dependent Ca^2+^-selective channel)	Manual	NA	13 Ba^2+^	20 ms single (resting block) vs 20 pulses at 1 Hz (use-dependent block) from -120 mV; amplitude unknown	Resting state block: 41 μM Use-dependent block: 10.4 μM	[32]
	HEK293	Ca_V_1.2, α_2_δ, β_2a_	Manual	~25ºC	1.8 Ca^2+^ 5 mM Ba^2+^	100 ms step from -90 to 10 mV at 0.03 Hz	250 ± 16 μM (Ca^2+^) 109 ± 12 μM (Ba^2+^)	[42]
Verapamil	Guinea pig ventricular myocytes	L-type Ca^2+^ current	Manual	RT, defined as 22–25ºC in	2 Ca^2+^	200 ms step from -50 to +10 mV at 0.2 Hz	0.16 ± 0.01 μM; n_H_ = 0.61	[41]
	hCav1.2 from Chantest	NA	IonWork Quattro	RT	NA	From -65 mV (applied for 10 s) a 500 ms depolarizing step to 0 mV was applied	2.69 μM, converted from pIC_50_ of 5.571	[43]; experimental details in [44]
	*Trichopulsia ni* cells (insect)	Ca_V_Ab (voltage-dependent Ca^2+^-selective channel)	Manual	NA	13 Ba^2+^	20 pulses at 1 Hz	475 ± 25 nM	[45]
	HEK293	Ca_V_1.2, α_2_δ, β_2a_ along with K_ir_2.3	Automated (PatchXpress 7000A) and manual	Automated 22–26ºC; manual 20–25ºC	30 Ba^2+^	100 ms step from -60 to +20 mV at 0.05 Hz	15 μM (automated) 47 μM (manual)	[39]
	Guinea pig ventricular myocytes	L-type Ca^2+^ current	Manual	20–25ºC	10 Ca^2+^	200 ms step from -70 to +10 mV at 0.2 Hz	0.79 μM	[39]
	CHO	hCa_V_1.2, β_2_, α_2_δ	Automated (QPatch and PatchXpress)	Ambient	1.8 Ca^2+^	150 ms pulse from -40 mV to 0 mV every 5 s	0.20 ± 0.02 μM; n_H_ = 0.80 ± 0.11	[33]
	CHO	Cav1.2, β_2_, α_2_δ_1_	IonWorks Barracuda	Ambient	7 Ca^2+^	2-pulse protocol: from -80 mV two 250 ms steps to +10 mV separated by 1s in between; Pre-conditioning protocol: from -80 mV step to -40 mV for 50 s then to +10 mV for 250 ms	Two-pulse protocol: Test pulse 1: 34.9 μM Test pulse 2: 9.8 μM Pre-conditioning protocol: 1.89 μM	[16]
	tsA-201 cells	Ca_V_1.2, α_2_δ, β_1b_	Manual	RT	10 Ba^2+^	100 ms pulses from -60 mV to +10 mV at 0.05 Hz	40.5 ± 0.9 μM	[10]
	HEK Ca_V_1.2	NA	Manual	Near physiological temperature	1.8 Ca^2+^	From -80 mV step to 0 mV for 40 ms then to +30 mV for 200 ms then ramp down to -80 mV within 100 ms (1.2 V/s); protocol was repeated at 0.2 Hz	0.206 μM*	[46]

*It is unclear whether the IC_50_ was measured at the 0 mV step or the ramp down phase in this study.

### Limitation, lessons learned, protocol standardization, and conclusion

There are several limitations in the present study that can impact drug potency estimation. The first is that the drug concentrations exposed to the recorded cell were not measured using an analytical method. Drug concentrations can deviate from the target concentration due to compound-specific factors and human errors. The former include nonspecific binding to the plastic and glass substrates within the patch clamp perfusion apparatus, potential insolubility at higher concentrations tested, and instability in the perfusion solution under the experimental condition. Notably, verapamil and diltiazem are compounds stated to be light sensitive (https://www.sigmaaldrich.com/US/en/sds/sigma/v4629) and advised to keep away from direct sunlight (https://documents.tocris.com/pdfs/tocris_msds/0685_sds.pdf?1646647276&_ga=2.29634103.924187291.1646647248-600272910.1596464753), respectively, on the Safety Data Sheet from their distributors. While verapamil solutions and stocks were protected from light in this laboratory, diltiazem was not, and time-dependent degradation for these as well as other compounds tested throughout the recording day cannot be ruled out. Human errors can also occur during drug stock and drug solution preparation. Concentration verification, if possible, should be included as a part of the study design to rule out the possibility that deviations of drug concentrations translate into variability in drug potency estimation. The second limitation is that Ca_V_1.2 current rundown under whole cell configuration could not be prevented, and current rundown was not corrected for drug potency estimation in this study. Although drugs were applied after the initial fast phase of rundown had ended, the IC_50_s may have still been underestimated. Given cell-specific rundown profile, if rundown correction were to be implemented, then accounting for individual cell’s rundown time course by fitting as many data points obtained in the control solution as possible, rather than using the time course plots derived from separate cells is recommended. Of note, although recordings in perforated patch configuration decrease cell dialysis-dependent rundown, this technique leads to higher R_s_ compared to whole cell recordings. Current rundown was a trade-off for voltage control in the present study, and the method to optimize voltage control was to use whole cell recording to obtain as small of R_s_ as possible followed by high degree of compensation. The third limitation is that R_s_ was not measured throughout the recordings. Not having this measure across time raises a logical concern that rundown of Ba^2+^ and Ca^2+^ current is due to large R_s_ changes. In this study, Ca^2+^ and Ba^2+^ currents recorded at near PT activate extremely fast, and large changes in R_s_ manifest as slowing of time-to-peak for the step current (for Ba^2+^ and Ca^2+^ currents) and shift in the voltage at which ramp peak occurred (for the Ba^2+^ current; Fig 3). Therefore, whether R_s_ changed dramatically or not during the control recording period was based on offline assessment of these current profiles. Progressive decreases in the current amplitude not accompanied by kinetic changes seem incompatible with the conjecture that rundown is secondary to large changes in R_s_. As the original electrophysiology records are available at https://osf.io/g3msb/, interested readers are encouraged to assess these files to draw independent conclusions regarding the mechanisms subserving rundown. The fourth limitation is that the reversal potentials of Ca^2+^ and Ba^2+^ currents were extrapolated from currents obtained between 0 and +20 mV steps (S5 Fig). These I-V relations were generated to assess adequacy of voltage control, inferred from graded increases in the current amplitudes to increasing voltages between -60 to -20 mV. For the purpose of measuring the reversal potential, extending the voltage steps to beyond the reversal potential would provide direct measurement for each cell. The fifth limitation is the uncertainty that the recorded Ca_V_1.2 current indeed reflects activity of channels with β_2_ and α_2_δ_1_ auxiliary subunits. The gray traces in Fig 1G and 1H show that the ratios of ramp-to-step current are quite variable across cells. Likewise, temperature sensitivity of the whole cell current was also quite different across cells (Fig 2E and 2J). Since auxiliary subunits modulate Ca_V_1.2 channel gating, it is possible that recordings in this study were from heterogenous Ca_V_1.2 channels with either one or both auxiliary subunits or channels in different states of phosphorylation.

A few lessons were learned by conducting the present study. First, data variability of Ca_V_1.2 channel block was collectively larger than those observed for cardiac hERG and Na_V_1.5 channel block based on this laboratory’s concurrent work. The concentration-inhibition plots presented in Figs 4 and 5 show variable degrees of current inhibition for individual cells to a given drug concentration, with no clear outliers observed. This level of data spread was not seen for hERG and Na_V_1.5 current inhibition by the same drugs [18]. Within-the-study data variability for Ca_V_1.2 current inhibition may be due to variable degrees of current rundown and heterogenous coupling between the channel α subunit with β_2_ and α_2_δ_1_ subunits, as the latter can also be targets of drugs. Second, state-dependent block of drugs on Ca_V_1.2 channels are common, inferred from the different IC_50_s obtained for the step and the ramp current. Understanding state-dependent block using voltage protocols that recapitulate cardiac AP may be important when trying to predict drug impact on cardiac electrophysiology. In the sinoatrial nodal cells, Ca_V_1.2 channels are activated rapidly upon membrane depolarization and contribute to the upstroke of the AP. Drug block of open Ca_V_1.2 channels (i.e., inhibition of the step current) may thus inform the potential of drug in affecting the heart rate and the PR interval. On the other hand, during a ventricular AP, Ca_V_1.2 channels are activated by the abrupt depolarization from rest, enter inactivated state during the plateau potential, and then become reactivated during the repolarizing phase of the AP before entering closed state. Information regarding how Ca_V_1.2 channel block develops during a ventricular AP, as well as drug effect on voltage-dependence of channel gating may inform potential change in AP shape (i.e., triangulation or simply shortening), thereby allowing better prediction of proarrhythmia risk. Some statistical [33] and *in silico* myocyte models have incorporated drug block of Ca_V_1.2 channels to assess the risk of *Torsade de Pointes* imposed by hERG channel block [34]. Incorporating IC_50_s measured at distinct current regions or accounting for state-dependent block of Ca_V_1.2 channels may lead to better model performance. The third lessons learned is that many drugs have n_H_ values much smaller than 1, and these values are dependent on the recording temperature and channel state (Fig 6). Once the contributions of current rundown and insolubility at higher tested concentrations to shallow concentration-inhibition graphs are ruled out, the most straightforward interpretation of these results is that drug-Ca_V_1.2 channel interactions leading to current inhibition are complex processes that may involve multiple binding poses (i.e., diltiazem), multiple binding sites (i.e., nitrendipine, roscovitine), or through allosteric mechanisms (i.e., diltiazem; naltrexone, Fig 7).

The present results showed that even with the same voltage protocol presented at the same stimulation rate, Cav1.2 pharmacology can still be sensitive to a variety of factors encountered during the experiments and during data analysis. When numerous experimental factors are different between two studies, as seen Crumb et al., 2016 [5] and Li et al., 2018 [6], drug potency estimates can be very different even for the same drugs. For Ca_V_1.2 data intended to support risk prediction or clinical interpretation, normalizing laboratory-specific practices is essential toward promoting data reproducibility across laboratories—a pivotal step toward engendering confidence amongst regulators for applying these *in vitro* data in the decision-making process. Toward this end, the FDA Cardiac Safety Studies Interdisciplinary Review Team (CSS-IRT) has posted a document regarding the recommended voltage protocols for cardiac ion channels, including Ca_V_1.2 channels, on its website (https://www.fda.gov/media/151418/download). The voltage waveform, stimulation frequency, compositions of the internal and Ca^2+^-based external solutions, and data analysis method are consistent with those used in the present study. For cardiac safety assessment, drug developers and regulators are following the guidelines released by the International Council for Harmonisation for Technical Requirements for Pharmaceuticals for Human Use: ICH S7B for nonclinical [35] and ICH E14 for clinical studies [36]. The newly released ICH E14/S7B Questions and Answers guideline offers best practice recommendations for patch clamp ion channel studies intended to support cardiac safety assessment [37] (ICH S7B Q&A 2.1), and the protocol used in this manuscript is consistent with these recommendations. ICH S7B Q&A 2.1 also recommends recording at near PT. This study tested two temperature controller models. Given the gravity-fed perfusion method and shallow bath chambers used here, the temperature controller model that heats the chamber bottom uniformly provided more stable temperature control. However, if a perfusion pump were used to maintain flow rate, then conceivably the temperature controller model that maintained bath temperature by heating the anodized aluminum platform would have also achieved stable temperature control. Experimenters interested in measuring drug block of Ca_V_1.2 channels at near PT are recommended to consider how bath temperature may fluctuate given the rig design, and importantly measure bath temperature near the recorded cell throughout the recordings to enable subsequent analysis of temperature fluctuations on within-the-study data variability. In conclusion, results from the present study offer rationale for the best practice recommendations regarding experimental design, conduct, and data quality consideration, and may benefit stakeholders considering utilizing Ca_V_1.2 channel data to support regulatory decision-making.

This table summarizes the IC_50_ and n_H_ values for Ca_V_1.2 channel block determined using the manual patch clamp method [5] and automated patch clamp system [6]. While these studies examined more overlapping drugs, only those for which Crumb et al., 2016 provided IC_50_ and n_H_ values are shown for comparison. The ratios are calculated as maximum vs. minimum IC_50_s. Crumb et al., 2016 used a Ca_V_1.2-CHO cell line from Cytocentrics Bioscience GmbH (Rostock, Germany), and did not provide information regarding subunits expressed. Experiments were conducted using whole cell patch clamp method at 36 ± 1ºC, and current was evoked using a rabbit ventricular AP waveform repeated at 10 s interval. Ba^2+^ (4 mM) was used as the charge carrier. The automated patch clamp data from Li et al., 2018 were generated using a CHO cell line that expressed hCa_V_1.2α, β_2_, α_2_δ_1_ subunits from Charles River Laboratories (Wilmington, MA). Recordings were performed using IonWorks Barracuda system operating in population perforated patch clamp mode. Recording temperature was not controlled and was expected to be higher than RT due to the heat produced during system operation. Inward current was evoked using the same “step-step-ramp” voltage waveform as used in the present study but repeated at 10 s interval. The current elicited by the 0 mV step was used to quantify drug effects. Ca^2+^ (6.8 mM) was used as the charge carrier. Compositions of the external recording solution were the same for both studies except for the charge carrier. For internal solution, Crumb et al., 2016 used 130 mM CsCl as the main salt, while Li et al., 2018 used 90 mM CsF + 50 mM CsCl. Current mediated by Ca_V_1.2 channels exhibit prominent rundown when recorded under the whole cell configuration. Percent current inhibition by drug reported by Li et al. 2018 was adjusted for current run down, using data from vehicle and positive control wells, even though recordings were obtained using perforated patch mode. Crumb et al., 2016 did not correct for current rundown nor specified the rate of rundown for the cells used. The predicted logP values for these drugs based on ChemAxon are provided as estimates of lipophilicity. The sources are as follows: bepridil (https://go.drugbank.com/drugs/DB01244), chlorpromazine (https://go.drugbank.com/drugs/DB00477), diltiazem (https://go.drugbank.com/drugs/DB00343), ondansetron (https://go.drugbank.com/drugs/DB00904), terfenadine (https://go.drugbank.com/drugs/DB00342), and verapamil (https://go.drugbank.com/drugs/DB00661). There is no relationship between the ratio of max-to-min IC_50_s and logP values for these drugs.

## Supporting information

S1 Fig I_passive_-subtraction vs. verapamil-subtraction. Recordings were obtained in external Ca^2+^. Cell ID was 18n20001 for panels **(A)** through **(C)**; 18n14007, panels **(D)** through **(I)**. Dashed lines in panels **(A)**, **(B)**, **(D)**, **(F)**, and **(H)** mark the 0 pA level. **A)** Original unsubtracted traces from a cell for which I_passive_ subtraction method worked well to quantify I_Ca-ramp_. Traces 1 and 35 were the 1^st^ and last recorded traces in control solution (black). Trace 85 was the last trace recorded in 30 nM tolterodine (light red). Trace 126 was the last trace recorded in 100 μM verapamil (red). I_passive_, calculated using R_input_ derived from trace 126, is shown in gray. Note the good alignment between I_passive_ and trace 126 at all voltages, suggestive of little to no endogenous voltage-dependent current in this cell under the specified experimental condition. **B)** I_passive_-subtracted traces show little to no outward current that is above 0 pA. I_Ca-ramp_ for this cell is quantified as the peak inward current during the voltage ramp down phase using I_passive_-subtracted traces. **C)** Time course plots of I_Ca-ramp_ (*top panel*), R_input_ (*middle panel*), and I_-80 mV_ (*lower panel*) for the same cell. **D)** Original unsubtracted traces from a cell for which verapamil subtraction method was used to quantify I_Ca-ramp_. Traces 1 and 62 were the 1^st^ and last recorded traces in control solution (black). Trace 114 was the last trace recorded in 3 μM tolerodine (medium red). Trace 143 was the last trace recorded in 100 μM verapamil (red). Note that this cell had larger outward current at the +30 mV step and the voltage ramp down phase than the cell illustrated above that was unmasked when the inward current was reduced (see medium red and red traces). **E)** *Top*, time course plots of absolute current amplitude, measured with a 20 ms window around the inward current peak (corresponding to ramp voltage 10.8 to -39.5 mV). Given the presence of outward current, as inward Ca^2+^ current was suppressed with verapamil, polarity of the absolute current reversed from being negative to positive. The middle and lower panels are time course plots of R_input_ and I_-80 mV_ for this cell. **F)** I_passive_-subtracted traces for the same cell showed that outward current remained. **G)** Voltages at which the maximal negative ramp current was identified from I_passive_-subtracted traces and by searching the entire voltage ramp down phase. Similar to panel **(E)**, there was a jump of voltage at which the maximal negative ramp current was identified when Ca^2+^ current was largely inhibited (i.e., ramp current approaching linear). **H, I)** Verapamil-subtracted traces for this cell **(H)** and time course plot of I_Ca-ramp_ quantified using verapamil-subtracted traces **(I)**.
(EPS)

S2 Fig Rundown of Ca^2+^ current for two additional cell lines. **A, B)** These panels show Ca^2+^ currents recorded from 2 additional cell lines. Currents were also mediated by hCa_v_1.2α, β_2_, and α_2_δ_1_ subunits expressed in HEK293 cells. Verapamil was not applied for cell in **(B)** since there was little Ca_V_1.2 current remaining after 150 traces. **C, D)** Summary time course plots of I_Ca-step_ **(C)** and I_Ca-ramp_ **(D)** recorded in control solution for the cell line represented by **(A)**. **E, F)** Summary time course plots of I_Ca-step_ **(E)** and I_Ca-ramp_ **(F)** for the cell line represented by **(B)**. Note that some cells did not last for all 200 traces of recording. Verapamil was not applied for this cell line.
(EPS)

S3 Fig Time course plots demonstrating amplitude fluctuations for I_Ca-0 mV_ but not I_Ca-ramp_. Recordings occurred in the control solution followed by bath application of 100 μM verapamil. Cell ID was 19322002. **A)** *Top*, current traces 100 and 114 (black) were obtained in the control solution; trace 200 (red), following steady state current inhibition by verapamil. I_passive_, shown in gray, was derived from the verapamil trace. *Bottom*, the voltage protocol used. **B)** The time course plots of I_Ca-0 mV_ and I_Ca-ramp_. **C**) R_input_ (*top*) and I_-80 mV_ (*bottom*).
(EPS)

S4 Fig Current-temperature relationship for additional cells shown in Fig 2E and 2J. Cells shown in panels **(A)** through **(E)** were recorded in 1.8 mM Ca^2+^; panels **(F)** through **(I)**, in 4.0 mM Ba^2+^. For each panel, the cell ID is shown on the top of the plots. **A)** This cell had 3 cycles of temperature manipulations and had rundown correction performed for both I_Ca-0 mV_ and I_Ca-ramp_. Fitting the rundown-corrected I_Ca-step_-temperature relation with a linear function yielded a slope of -0.0405 nA/°C and a Y-intercept of 0.704 nA. No relation was observed between I_Ca-ramp_ and temperature. **B)** This cell had 5 cycles of temperature manipulations and did not require rundown correction of the current amplitudes. The slope of the I_Ca-0 mV_-temperature relation was -0.121 nA/°C, and the Y-intercept was 3.406 nA. No relation was observed between I_Ca-ramp_ and temperature. **C)** This cell had 5.5 cycles of temperature manipulations and did not require of rundown correction of the current amplitudes. The slope of the I_Ca-step_-temperature relation was -0.0278 nA/°C, and the Y-intercept was 0.342 nA. I_Ca-ramp_ was also inversely related to temperature for this cell. The slope of the I_Ca-ramp_-temperature relation was -0.0160 nA/°C, and the Y-intercept was 0.0694 nA. **D)** This cell had 2 cycles of temperature manipulations and did not require rundown correction of current amplitudes. The slope of the I_Ca-step_-temperature relation was -0.129 nA/°C, and the Y-intercept was 2.66 nA. No relation was observed between I_Ca-ramp_ and temperature (r = 0.27). **E)** This cell had 2 cycles of temperature manipulations and did not require rundown correction of current amplitudes. The slope of I_Ca-0 mV_-temperature relation was -0.288 nA/°C, and the Y-intercept was 8.059 nA. I_Ca-ramp_ was also inversely related to the bath temperature for this cell. The slope of the I_Ca-ramp_-temperature relation was -0.0301 nA/°C, and the Y-intercept was 0.808 nA. **F)** This cell had 4 cycles of temperature manipulations. Rundown correction was performed for both I_Ba-step_ and I_Ba-ramp_. The slope of rundown corrected I_Ba-step_-temperature relation was -0.117 nA/°C, and the Y-intercept was 1.414 nA. I_Ba-ramp_ appeared to be inversely related to the bath temperature. The slope of rundown corrected I_Ba-ramp_-temperature relation was -0.0292 nA/°C, and the Y-intercept was -0.771 nA. **G)** This cell had two cycles of temperature manipulations. Rundown correction was performed on both I_Ba-0 mV_ and I_Ba-ramp_. The slope of the I_Ba-0 mV_-temperature relation was -0.146 nA/°C, and the Y-intercept was 2.360 nA. I_Ba-ramp_ appeared to be inversely related to the bath temperature. The slope of I_Ba-ramp_-temperature relation was -0.0225nA/°C, and the Y-intercept was -0.558 nA. **H)** This cell had 5 cycles of temperature manipulations and did not require rundown correction of current amplitudes. The slope of the I_Ba-step_-temperature relation was -0.120 nA/°C, and the Y-intercept was 3.165 nA. I_Ba-ramp_ was also inversely related to the bath temperature. The slope of the linear fit was -0.0603 nA/°C, and the Y-intercept was 1.426 nA. **I)** This cell had 5 cycles of temperature manipulations and did not require rundown correction of current amplitudes. The slope of I_Ba-step_-temperature relation was -0.287 nA/°C, and the Y-intercept was 7.098 nA. I_Ba-ramp_ was also inversely related to the bath temperature. The slope of the I_Ba-ramp_-temperature relation was -0.0770 nA/°C, and the Y-intercept was 0.774 nA.
(EPS)

S5 Fig Normalized I-V relation of Ba^2+^ and Ca^2+^ current. Currents were evoked from a holding potential of -80 mV to +20 mV in external Ba^2+^ **(A)** or +15 mV in external Ca^2+^ **(B)** with 5 ms voltage steps in 5 mV increments. For each cell, the peak current evoked by each voltage step was normalized to the maximum current recorded. The normalized and averaged current from all cells for a particular voltage step was then plotted against that membrane voltage to generate the normalized I-V plots. Reversal potentials (E_rev_) of the Ba^2+^ and Ca^2+^ currents were estimated by fitting the averaged data points from -5 mV to +20 mV in external Ba^2+^ **(A)** or -5 mV to +15 mV in external Ca^2+^ **(B)** with linear functions in the form of y = a + bx, and then solving for x when y = 0. For **(A)**, a = 0.794; b = -0.023. For **(B)** a = 0.799; b = -0.017.
(EPS)

S6 Fig Exemplar time course plots of Ca^2+^ current recorded at near PT following applications of: A) buprenorphine, B) norbuprenorphine, C) methadone, D) naltrexone, E) naloxone, and F) tolterodine. Open black symbols reflect absolute current amplitudes obtained by analyzing unsubtracted current traces; open red symbols reflect I_passive_-subtracted current amplitudes. All plots show absolute and I_passive_-subtracted amplitudes. Due to current rundown, data points for earlier traces for some cells are off the scale hence not illustrated.
(EPS)

S7 Fig Exemplar time course plots of Ba^2+^ current recorded at near PT following applications of: A) buprenorphine, B) norbuprenorphine, and C) naloxone. Open black symbols reflect absolute current amplitudes obtained by analyzing unsubtracted current traces; open red symbols reflect I_passive_-subtracted current amplitudes. All plots show absolute and I_passive_-subtracted amplitudes. Due to current rundown, data points for earlier traces for these cells are off the scale hence not illustrated.
(EPS)
