# Supplementary material for: Experimental factors that impact CaV1.2 channel pharmacology—Effects of recording temperature, charge carrier, and quantification of drug effects on the step and ramp currents elicited by the “step-step-ramp” voltage protocol
Source: PLoS One. 2022 Nov 23;17(11):e0276995. doi: 10.1371/journal.pone.0276995 (PMC9683570; doi:10.1371/journal.pone.0276995)
Supplement: Attachment — Submitted filename: Response to Reviewers.docx [file pone.0276995.s009.docx]

Wendy W. Wu

DARS/CDER/FDA

10903 New Hampshire Avenue

Silver Spring, MD 20993

Tel: 301-796-0081

Email: wendy.wu@fda.hhs.gov

**Reviewer #1: --- Overall ---**

**The authors have provided a very detailed “response to reviewers”, which has clarified the concerns that I had about the study overall and helped strengthening a positive opinion on my part.**

**In going through the resubmitted version of the manuscript, the combination of results and discussion has greatly improved readability. Neophyte readers will greatly appreciate Table 2 as well.**

We thank Reviewer 1 for these encouraging remarks.

**--- Minor ---**

**Perhaps this is a personal point of view, but I feel that some of the detail provided in the “response to reviewers” has not been incorporated to the manuscript, and doing so could still add further value. Specifically,**

**(1) If the authors did not observe clear outliers in their data despite high cell-to-cell variability, please state so in the manuscript.** This statement is now included in the manuscript. Please see lines 656 - 657.

**(2) If the authors found that literature information or empirical evidence are insufficient to address a certain question (e.g. specific recommendations about temperature control?) please state so in the manuscript. Stating that information is insufficient is more helpful to readers, at least in my opinion, than stating nothing.** Please see lines 704 – 714. We have stated our experiences using the two temperature controllers, emphasizing that for gravity-fed perfusion systems (that could lead to changes in the flow rate due to changing solution levels in the reservoirs) and small/shallow bath chambers like ours, the model that heats the chamber bottom (on which the coverslip with cells directly sat) provided more stable bath temperature maintenance near PT. As our temperature fluctuations were related to changes in the flow rate, we have also speculated that the other temperature controller model may have worked well should a perfusion pump be used to deliver the recording solutions. Finally, we have provided two recommendations for experimenters interested in testing Ca_V_1.2 pharmacology at near PT: 1) to understand the extent of temperature fluctuations per their rig designs, and 2) to capture temperature readout throughout the recordings (to enable offline analysis of temperature sensitivity should one be interested).

We have revised the manuscript by incorporating Reviewer 1’s two specific requests mentioned above. Reviewer 1 (and 2 and 3) had raised many important questions that we have addressed in the cover letter accompanying the first revision. However, as was pointed out then, the amount of information provided in this study already made the manuscript dense, which Reviewer 1 cautioned would discourage readers. We are concerned that by incorporating all the responses in the first cover letter into the second revision, we would further exacerbate the issue, especially given that addressing Reviewer 3’s two concerns in revision 2 also increased text and introduced new concepts. *PLOS ONE* publishes questions raised during the review process as well as authors’ point-to-point responses. Therefore, interested readers will have access to all the Q&As. We will also upload both cover letters to <https://osf.io/g3msb/> associated with this manuscript.

**Reviewer #3: Although it is obvious that this work required extensive time commitment and the Authors clearly spent time and effort to address all concerns in their response and revision, the following serious concerns remain.**

**1. In most sample traces (e.g., Suppl Fig 6 and 7) the current run down is unusually strong (especially for Ba, for which most investigators find much less if at all run down over time). In a number of example traces, the run down is very variable over the time course. This variability and the very strong run down seems to us to preempt quantification of drug effects, which appear often smaller than the observed run down that is intrinsic to the authors’ measurements. One possibility is that this is due to a strong change of Rs over time. Because no time courses are presented for Rs, this possibility cannot be excluded.**

We thank Reviewer 3 for this comment. The extent of Ca^2+^ and Ba^2+^ current rundown is cell-specific, and can be extensive as we have pointed out in the manuscript. We agree that this phenomenon is not anticipated *a priori* based on literature search, hence we tested 3 cell lines total with Ca_V_1.2 channels that included β_2_ and α_2_δ_1_ subunits (see Sup. Fig. 2 for two additional cell lines). That IC_50_ could be underestimated due to rundown was stated in the original manuscript (lines 620 – 622). We believe that current rundown as well as its potential impact on estimating drug block potencies of Ca_V_1.2 channels should be conveyed clearly and discussed, as this phenomenon should be considered when interpreting Ca_V_1.2 data.

We acknowledge that not having a time course plot of R_s_ throughout the recording for every cell is a limitation, and have included this point in the manuscript (see lines 629 – 640). In our previous study on Na_V_1.5 current, we measured R_s_ for every trace (Wu et al., 2019; <https://pubmed.ncbi.nlm.nih.gov/31255744/>). However, estimating and compensating R_s_ in between each trace took time, and doing so would have precluded our delivering voltage waveform at 5 s intervals (a recommendation of the ICH S7B Q&A 2.1 is to not miss frequency-dependent block, which would be a concern for pharmacology experiments if stimulating at an even slower frequency than the present study). Another option is to the pause recording during the middle of the experiment to obtain R_s_ measurement. However, interruption of pacing would then lead to a change in the current amplitude, thereby prolonging the recording time in the control solution. Ca^2+^ and Ba^2+^ currents recorded at near PT activate extremely fast, and a large change in R_s_ can be seen as a slowing of time-to-peak for the step current and a shift in the voltage at which ramp peak occurred (also see Fig. 3). Below are Ba^2+^ current traces (65, 75, 85, 95, and 105) from the buprenorphine cell shown in Supplemental Fig. 7, illustrating no change in the time-to-peak for the step current as well as the voltage at which the ramp peak occurred in spite of current rundown.

We do not believe current rundown, including that seen in Ba^2+^ experiments, was due to strong and progressive change in R_s_ over time based on these offline analyses. Having said that, we thank Reviewer 3 for raising this logical concern. By making all electrophysiology records available through <https://osf.io/g3msb/>, we provide interested readers the opportunity to go over the original recordings to form independent conclusions, including mechanisms subserving rundown.

**2. Reversal potentials were not determined by testing over the whole voltage range but extrapolated from currents obtained for depolarizations between 0 and +20 mV (Suppl Fig 5). It is unclear why the reversal potentials were not directly measured. Also critically, for Ba should be closer to 50 mV and Ca closer to 80 mV than rather than 35 and 46, respectively.**

We thank Reviewer 3 for this comment, and have now included our extrapolation method for the reversal potentials of the Ba^2+^ and Ca^2+^ currents as a limitation (see lines 640 – 645). In these experiments, the I-V relations were generated for a subset of cells to assess voltage control, by verifying that the currents increased in an incremental fashion and not “jump” to the maximum amplitude. Our focus was thus in the voltage range between -60 and -20 mV. We agree that for the purpose of assessing reversal potential, extending the voltage steps to beyond the reversal potential would offer direct measurement.
